# Bioinspired Microtexturing for Enhanced Sweat Adhesion in Ion-Selective Membranes

**DOI:** 10.34133/cbsystems.0337

**Published:** 2025-08-05

**Authors:** Marc Josep Montagut Marques, Takayuki Masuji, Mohamed Adel, Ahmed M. R. Fath El-Bab, Kayo Hirose, Kanji Uchida, Hisashi Sugime, Shinjiro Umezu

**Affiliations:** ^1^ Department of Integrative Bioscience and Biomedical Engineering, Waseda University, Tokyo, Japan.; ^2^ Department of Modern Mechanical Engineering, Waseda University, Tokyo, Japan.; ^3^ Mechanical Engineering Department, Helwan University, Cairo, Egypt.; ^4^ Department of Mechatronics and Robotics Engineering, Egypt-Japan University of Science and Technology (E-JUST), Alexandria, Egypt.; ^5^ Department of Anesthesiology and Pain Relief Center, The University of Tokyo Hospital, Tokyo, Japan.; ^6^ Department of Applied Chemistry, Kindai University, Osaka, Japan.

## Abstract

Advancements in health wearable technology hold the potential to prevent critical health issues such as hyponatremia and other hydration-related conditions often triggered by intense physical activities. Approaches to address this issue include the development of thin-film wearable sensors incorporating carbon nanotubes (CNTs), which offer scalability, lightweight design, and exceptional electrical properties. CNT paper serves as an ideal substrate for electrochemical sensors like ion-selective membranes (ISMs), enabling effective on-skin electrolyte monitoring. However, current on-skin devices often face limitations in maintaining performance during human motion. This study introduces a bioinspired surface texturing technique that mimics the microstructures of rose petals to enhance wettability, self-cleaning, and ISM sensitivity. By replicating the mechanical properties of the surface texture found on rose petals, the newly developed ISM achieves accurate measurements across a 2-mm air gap, offering an improved interfacing solution that promotes better sweat recirculation and comfort. This advancement overcomes the constraints of traditional sensors, paving the way for more reliable and effective noninvasive health monitoring in real-world conditions.

## Introduction

Thin-film sensors represent a transformative approach to overcoming the challenges associated with human motion in wearable health technologies. Their development is driven by the need for lightweight, flexible, and adaptable electronic systems that seamlessly integrate with the human body [1–3].

The integration of thin-film materials, such as carbon nanotubes (CNTs) and ion-selective membranes (ISMs), has demonstrated exceptional performance in the development of adaptable electrochemical sensors in the field of health wearables. CNTs exhibit unique mechanical flexibility, making them ideal for skin-mounted applications, where user comfort and reliable signal acquisition are essential [4–7]. Furthermore, the superior electrical conductivity of CNTs enhances their effectiveness as signal transducers when combined with ISMs in electrochemical sensing platforms [8–10]. ISMs, widely utilized for the selective detection of metallic ions in sweat, provide a noninvasive approach to monitoring critical physiological parameters, offering valuable insights into electrolyte balance and overall health [11,12]. Bandodkar et al. [13] pioneered on-skin sweat sensing with their sodium detecting epidermal tattoo [2], a milestone that spurred widespread efforts to miniaturize electronics and develop multi-parametric wearables for comprehensive health diagnostics [14–17].

Sodium concentration in sweat has been shown to be a key indicator of hydration and muscle performance, as highlighted by Baker [12]. Electrolyte loss through sweating can lead to dehydration, which over time contributes to conditions such as kidney stones, fatigue, and muscle pain [18,19]. Dehydration often goes unnoticed until symptoms emerge, as reported by Shaheen et al. [20], and the brain’s natural response may not be rapid enough to maintain hydration, particularly in demanding or distracting situations [21]. Studies show that up to 35% of hospital patients suffer from hyponatremia [19], an electrolyte imbalance disorder. Severe cases of hyponatremia can result in medical emergencies. The conditions highlight the critical need for wearable technologies capable of real-time sodium monitoring. Emerging commercial products for hydration diagnostics underscore this growing demand [18].

While ISMs offer a pathway to real-time electrolyte monitoring, their inherent hydrophobicity presents challenges in obtaining the required sweat samples [22–24]. Environmental variables, such as body motion and friction, often interfere with sensor-to-skin readouts, necessitating tightly constrained interfaces to maintain performance [25,26]. This limitation is often addressed by creating tight constraints between the skin and the device [27–32], and relaying on capillary flow. Adhesives may be suitable for short-term monitoring, but their long-term use presents clear disadvantages to skin health, including sweat stagnation, bacterial growth, infections, and other risks related to excess sweat [33,34], which can lead to conditions such as dermatitis [35] or skin allergies [36]. Adhesive-based wearables have limited access to the sensing area, making it difficult to recalibrate sensors once applied to the skin. For all the reasons mentioned above, the development of noncontact devices is crucial for improving sensor performance, ease of use, and long-term reliability.

To address the challenges of traditional electrochemical sensors, we developed a bioinspired, microtextured ISM that enhances wettability and self-cleaning properties, improving the interface between the sensor and skin. Enhancing the wettability increased the sensing range of the microchannel from 300 [37] to 2,000 μm, marking a milestone toward noncontact sensing. Rose petals exhibit a unique wetting behavior: They are hydrophilic when in contact with small amounts of water, but become hydrophobic once the water exceeds a certain threshold, triggering a self-cleaning effect [38]. This distinctive property inspired the design of our sensors, where the rose petal’s dual wetting behavior was successfully replicated to enhance functionality.

The ISM incorporates bioinspired rose petal textures that optimize interaction for sweat collection and recirculation, enhancing its functionality in both wearable and integrated systems. This design enables precise and stable electrolyte detection, even during high-motion activities, by dynamically regulating surface tension based on water load. Its ability to maintain consistent hydration and signal stability makes it highly suitable for integration into prosthetics, exoskeletons, and human–machine interfaces, where real-time biochemical monitoring can enhance adaptive control, user comfort, and system responsiveness. The resulting system offers both qualitative and quantitative advantages over conventional ISM configurations, ensuring reliable performance in dynamic and high-mobility environments.

## Methods

### Materials

Multi-walled CNTs (50 to 90 nm diameter, 5 to 20 μm length, >95% carbon basis, chemical vapor deposition) and isopropyl alcohol were obtained from Merck KGaA (Japan) for fabricating carbon-based electrodes. A Durapore membrane [polyvinylidene difluoride (PVDF), 0.45 μm pore size, 47 mm filter diameter] was also sourced from Merck KGaA. Ag/AgCl conductive ink from BAS Inc. (Japan) was used for the reference electrode and contact fabrication.

Sodium ionophore X, sodium tetrakis[3,5-bis(trifluoromethyl)phenyl]borate, poly(vinyl chloride) (PVC; high molecular weight), bis(2-ethylhexyl) sebacate (DOS), and tetrahydrofuran (THF) were procured from Merck KGaA for sodium ISM synthesis.

SYLGARD 184 polydimethylsiloxane (PDMS) was used to create bioinspired molds, with *Rosa rubiginosa* (Pink Antique Explorer rose petals) sourced from a local flower store in Tokyo for biomimetic texture replication. All chemicals were used as received without further purification.

### Membrane synthesis with bioinspired texturing

The carbon nanotube forest (CNTF) sponge was fabricated by dispersing CNTs (0.1 mg/ml) in isopropyl alcohol using an ultrasonic stirrer (18 kHz) for 10 min. This solution was filtered through a Durapore membrane under vacuum, and the sponge was dried at >150 °C for 1 h to enhance mechanical stability. The sponge was cut into 2 mm × 10 mm strips for electrode preparation. Ag/AgCl ink was applied to the CNTF tips using the doctor blade method, with a precision-cut paper mask ensuring localized deposition.

For bioinspired texturing, molds of rose petal surfaces were prepared to replicate the hydrophilic patterns of the petal’s exterior (side A) and interior (side B). Rose petals were pressed flat onto glass slides and encased in a polymethyl methacrylate (PMMA) frame (15 mm × 15 mm × 3 mm). PDMS was poured into the frame, de-bubbled under vacuum for 30 min, and cured at 60 °C for 2 h according to the manufacturer’s specifications. After curing, the molds were peeled off to reveal the petal surface relief.

The sodium ISM was synthesized by mixing 2 mg of sodium ionophore X, 11 mg of sodium tetrakis[3,5-bis(trifluoromethyl)phenyl]borate, 66 mg of PVC, 130 mg of DOS, and 1.17 g of THF. The mixture was homogenized at 5,600 rpm for 5 min with a vortex mixer, followed by 30 min of ultrasonic stirring at 18 kHz. The solution was spin-coated onto the rose-petal-inspired molds at 1,500 rpm for 15 s to create a homogeneous membrane surface of approximately 100 μm. After solvent evaporation, the membrane was carefully peeled off with carbon tweezers. A 2 mm × 2 mm section was excised and affixed to a CNTF strip using a small amount of the precursor solution.

### Surface characterization

Water droplet contact angle (CA) tests were performed to evaluate hydrophilicity. The tested materials included Ag/AgCl, CNTF sponge, and various ISM variations. Tests were conducted using ~10-μl tap water droplets in face-up, face-down, and lateral orientations relative to the ground plane. Droplet profiles were captured using a digital microscope against a high-contrast background.

The surface morphology of rose petals, PDMS molds, and ISMs was examined via scanning electron microscopy (SEM) to assess the fidelity of biomimetic replication. Feature dimensions were measured and analyzed using ImageJ software. Submicrometer features, such as ripples, were measured at ×15,000 magnification, while broader features, such as isles, were measured at ×1,000 magnification. The electron beam was set at 20 kV.

### Electrochemical sensing characterization

Electrochemical properties of the ISM were characterized using a potentiostat (ECstat 101, Frontier) in open-circuit potential (OCP) mode. In this configuration, the reference and counter electrodes were combined, and an Ag/AgCl electrode served as the common electrode. Sensitivity tests for sodium ion detection were performed by exposing the sensor to sodium chloride (NaCl) solutions in deionized (DI) water at concentrations of 0, 10^−3^, 10^−2^, and 10^−1^ M. Each concentration was measured for 600 s to ensure signal stabilization and reproducibility. Selectivity was evaluated by introducing equivalent molarities of potassium chloride (KCl) solutions and comparing the resulting potential changes to assess the sensor’s ability to differentiate between sodium and potassium ions. All measurements were conducted in a controlled environment at room temperature (25 °C) to minimize the influence of external variables. The sensor was preconditioned in 10^−2^ M NaCl solution for 12 h prior to testing to ensure optimal ion exchange performance. Data were normalized relative to the stabilization point at 0 M NaCl concentration to facilitate comparative analysis. Additionally, the Nernst equation and the Nikolskii–Eisenman formalism were applied to analyze the theoretical and experimental sensitivity, providing a deeper understanding of the sensor’s electrochemical behavior. To ensure the reliability of the results, 3 independent sensors were tested, with a total of 15 replicates performed for each concentration. Statistical analysis, including standard error and linear regression, was utilized to evaluate variability and quantify the sensor’s performance metrics.

### Adhesion and self-cleaning characterization

To evaluate the water adhesion and self-cleaning properties of the rose petal and the ISM, 4 distinct experiments were conducted.

Maximum water load capacity: Surfaces were oriented face-down on a precision scale. A single water droplet was deposited onto the surface using a microsyringe and incrementally filled until droplet collapse occurred. The maximum recorded weight indicated the surface’s maximum load capacity. The procedure from the first test was repeated, but surfaces were oriented at a 90° angle to assess how gravity and surface orientation impacted adhesion properties.

Self-adhesion measurement: Self-adhesion was defined as the ability of a surface to lift water upon contact with a wetted substrate. The test involved weighing the surface before and after contact with a water-coated platform. The adhered water was observed and photographed using a digital microscope, with light reflections used to quantify the extent of water retention.

Dynamic adhesion: Dynamic adhesion was assessed by measuring the retention of water droplets under controlled motion. Two sets of experiments were performed; water droplets weighing 15 mg and 10 mg were placed on the test surface. The surface, along with a 6-g load, was released from a height of 5 cm using a custom 3-dimensionally (3D) printed structure to ensure a controlled and repeatable free-fall. Following the fall, the remaining water load on the surface was recorded. The test was repeated in cycles until the retained water weight fell below 1 mg. This threshold marked the final repeatability cycle, providing insights into the durability and dynamic adhesive performance of the surface under motion.

Recirculation and self-cleaning: ISMs were integrated into a 3D-printed channel structure simulating the gap between human skin and the sensor. Air gap sizes of 0.5, 1, 1.5, and 2 mm were tested. Sodium ion solutions of varying concentrations and DI water were sequentially flowed through the channels to monitor real-time signal acquisition and evaluate the membranes’ responsiveness to changes in ionic concentration under simulated on-wearable conditions.

### Wearable device fabrication

Three wearable device designs were tested to channel sweat to the sensing area.

PDMS design: A hard resin mold was created using a digital light processing (DLP) 3D printer. The design of the mold included microfluidic channels and a passive pump capable of recirculating sweat using the movement of the body. PDMS was poured into the mold, de-bubbled under vacuum for 30 min, and cured at 60 °C for 2 h. The cured structure was demolded. A layer of Tegaderm (TM) was then used to seal the microchannel structures.

Flexible resin design: Fabricated using a liquid crystal display (LCD) 3D printer and a flexible, clear ultraviolet (UV) resin. The device was printed with varying air gaps to test the effectiveness of sweat collection.

Hard resin design: Made from a hard, gray UV resin using an LCD 3D printer, following the same design as the flexible resin variation.

Rubber straps were attached to fitting holes in all designs for comfortable wearability.

### Real-time on-body sweat analysis

The wearable device was securely fastened to the upper wrist of the subject to evaluate its performance in real-time sweat analysis during a controlled treadmill exercise. The test area on the arm was thoroughly cleaned with an alcohol swab before device placement to ensure optimal sensor–skin interface. The treadmill was set to a constant speed of 8 km/h, and the running session lasted approximately 20 min, creating conditions conducive to sustained sweat production. Prior to the experiment, the ion-selective sensors were calibrated using standard NaCl solutions to establish accurate baseline measurements. During the exercise, the sodium ion concentration (Na^+^) in sweat was continuously monitored using a potentiostat, and real-time data were recorded. To assess the sensor’s response time and repeatability, DI water was applied to the skin at 950 and 1,300 s, simulating sweat recirculation and ensuring that the sensor responded effectively to changes in ionic concentration. The device’s performance was evaluated based on its ability to maintain stable signal acquisition despite motion-induced disturbances. A 2-mm sensing channel gap was used to assess the sensor’s capability for noncontact operation, and results demonstrated reliable adhesion and consistent signal output during the session. Data analysis included response time calculations, signal stability assessment, and calibration threshold verification, highlighting the sensor’s robustness and applicability in dynamic environments.

### Statistical analysis

For the evaluation of sensor sensitivity, the slope of the calibration curve, representing the sensitivity, was calculated using linear regression, and the coefficient of determination (*R*^2^) was determined to evaluate the linearity of the sensor’s response. The limit of detection (LOD) and limit of quantification (LOQ) were calculated using the standard deviation of the baseline signal and the slope of the calibration curve, defined as LOD = 3*σ*/*m* and LOQ = 10*σ*/*m*, where *σ* is the standard deviation of the blank signal and *m* is the slope.

To characterize CAs, 50 droplets were applied to each surface, and the average CA was calculated for each sample. The maximum load capacity was assessed by averaging measurements from 100 droplets for each case, including the self-adhesion experiment. The self-adhesion data were further analyzed using the 5-number summary method to evaluate the probability of optimal adhesion under varying conditions.

For the evaluation of microdistances, SEM images of the features were analyzed using ImageJ software. Measurements were taken from 200 distinct areas per feature to ensure statistical robustness. The distribution of variations across these features was analyzed using the 5-number summary method, implemented via the Matplotlib library.

The self-cleaning property was characterized using linear regression to assess the repeatability of the experiments by analyzing the slope of the regression line. The slopes for each sensor variation and channel depth were calculated and averaged. Standard error analysis was conducted to identify instances of poor sensor interface performance, providing additional insights into the variability of self-cleaning behavior. These statistical analyses were performed using the Scikit-learn Python module, with no additional data adjustments beyond the procedures described above. This comprehensive statistical approach ensures accurate characterization of the sensor’s performance and provides insights into key functional properties.

## Results and Discussion

### Surface characterization

**Fig. 1.**
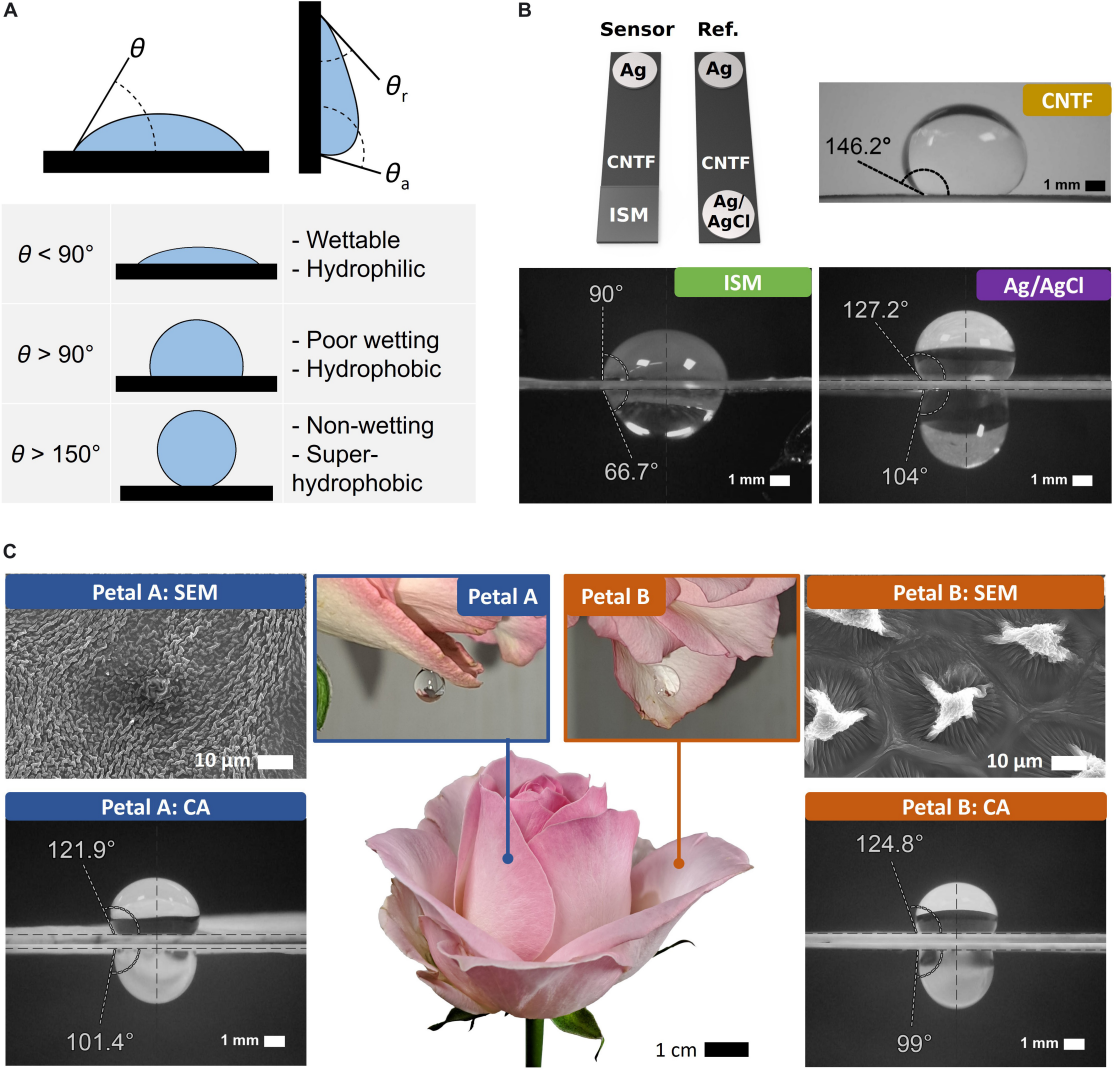
Contact angle (CA) characterization of sensor materials. (A) Schematic of CA definitions: *θ* (surface parallel), *θ*_a_ (advancing), and *θ*_r_ (receding) on a 90° inclined plane. CAs provide insights into hydrophilicity and wettability. (B) Sensor materials: CNTF sponge (CA 146.2°) is highly water-repellent; unmodified ISM (CA 90°) shows partial wetting; reference electrode (CA 127.2°) is hydrophobic but mimics rose petal adhesion. (C) Rose petal analysis: Side A has 1-μm wrinkles with submicrometer gaps; Side B features 8-μm spikes in a 30-μm honeycomb pattern. Despite hydrophobic CA, hysteresis highlights complex wetting behavior.

To understand the water-interfacing properties of the materials forming the device, we first established the definition of wetting and hydrophilic surface using static CA measurements, following Kock–Yee law [39] and Giridhar et al. [40] (Fig. 1A). However, this classification assumes a static surface with water facing upward and does not account for dynamic environments. As per Bharat et. Al [41], CA hysteresis and surface cavities—causing the Cassie-Baxter or Wenzel effects—are necessary to understand water adhesion at an angle. Therefore, CA and hysteresis were measured under face-up, face-down (Fig. 1B), and lateral conditions. The results are summarized in Table 1 and Supplementary Information S1.

**Table 1.** Water droplet contact angles

*n* = 50	CNTF	Ag/AgCl	Petal A	Petal B	Surface A	Surface B	Surface C (*N*)
15-μl droplet							
Face up *θ*/(°)	146.2	127.2	114.9	109.4	76.8	105.8	90
Face down *θ*/(°)	-	104	101.4	99	66.6	95.3	66.7
Ratio (down/up)	-	0.82	0.9	0.9	0.86	0.9	0.74
Perpendicular *θ*_a_ /(°)	-	128.1	124	110.8	95.7	100.8	100.1
Perpendicular *θ*_r_ /(°)	-	68.4	74	68.3	50.4	55.7	60
CA hysteresis	-	0.99	0.83	0.72	0.74	0.75	0.68

Construction materials were characterized to assess their potential impact on the performance of the sensor. The CNTF electrode substrate presented a CA of 146.2°, classifying it as superhydrophobic. Due to its lack of adhesion, face-down experiments were not feasible, as water droplets could not adhere to the surface. This classification aligns with previous findings, such as those reviewed by Yong et al. [42], confirming the superhydrophobic properties of CNTF materials. The reference Ag/AgCl ink electrode was fabricated by the blade-coating method onto the tip of the substrate strip, using a paper mask. The reference electrode exhibited a CA of 127.2°, categorizing it as highly hydrophobic. Despite its hydrophobicity, the surface demonstrated strong adhesion when inverted, as shown in Fig. 1B, allowing the electrode to be effectively wetted for signal acquisition, ensuring stable and reliable measurements.

The ISM was characterized to assess the poor wetting observed during sample measurements. Fabricated via drop-casting, the ISM membrane forms passively under gravity, resulting in a flat surface. The resulting membrane exhibited a CA of 90°, indicating incomplete wetting. During sensitivity tests, the electrode resisted soaking when approached to water, requiring full immersion to achieve reliable data acquisition. This behavior poses challenges when trying to measure mediums with limited constraints, such as on-skin sweat, and is commonly solved by directly attaching sensors to the skin, restricting sweat acquisition to the capillary flow.

To extend the application of ISMs in dynamic environments, we examined how natural specimens interact with water, bamboo, *Phragmites australis*, Pink Antique Explorer, and *Dianthus caryophyllus*, were initially characterized based on review studies [43,44]. The rose petal was identified as an ideal biological example due to its dual ability to retain and self-clean water droplets, providing versatile behavior. The wetting behavior of both sides of the petal was characterized: side A (outer-facing) and side B (inner-facing), as shown in Fig. 1C. Both sides exhibited hydrophobic properties, with CAs of 121.9° (side A) and 124.8° (side B). Despite their hydrophobicity, the surfaces demonstrated water retention when oriented face-down and laterally. Ag/AgCl-coated surfaces presented an adhesion similar to the rose-petal surface; however, the self-cleaning characteristic was not observed. Self-cleaning was observed when larger water droplets exceeded the petal’s retention capacity. The surface transitioned to a self-cleaning state, eliminating the water load as a whole and leaving no moisture. This unique combination of adhesion and self-cleaning inspired the design of the microtextured surface for enhanced sensor functionality in sweat analysis applications. This phenomenon is later explained in the hydrodynamic properties of the sensor.

### Bioinspired membrane fabrication

**Fig. 2.**
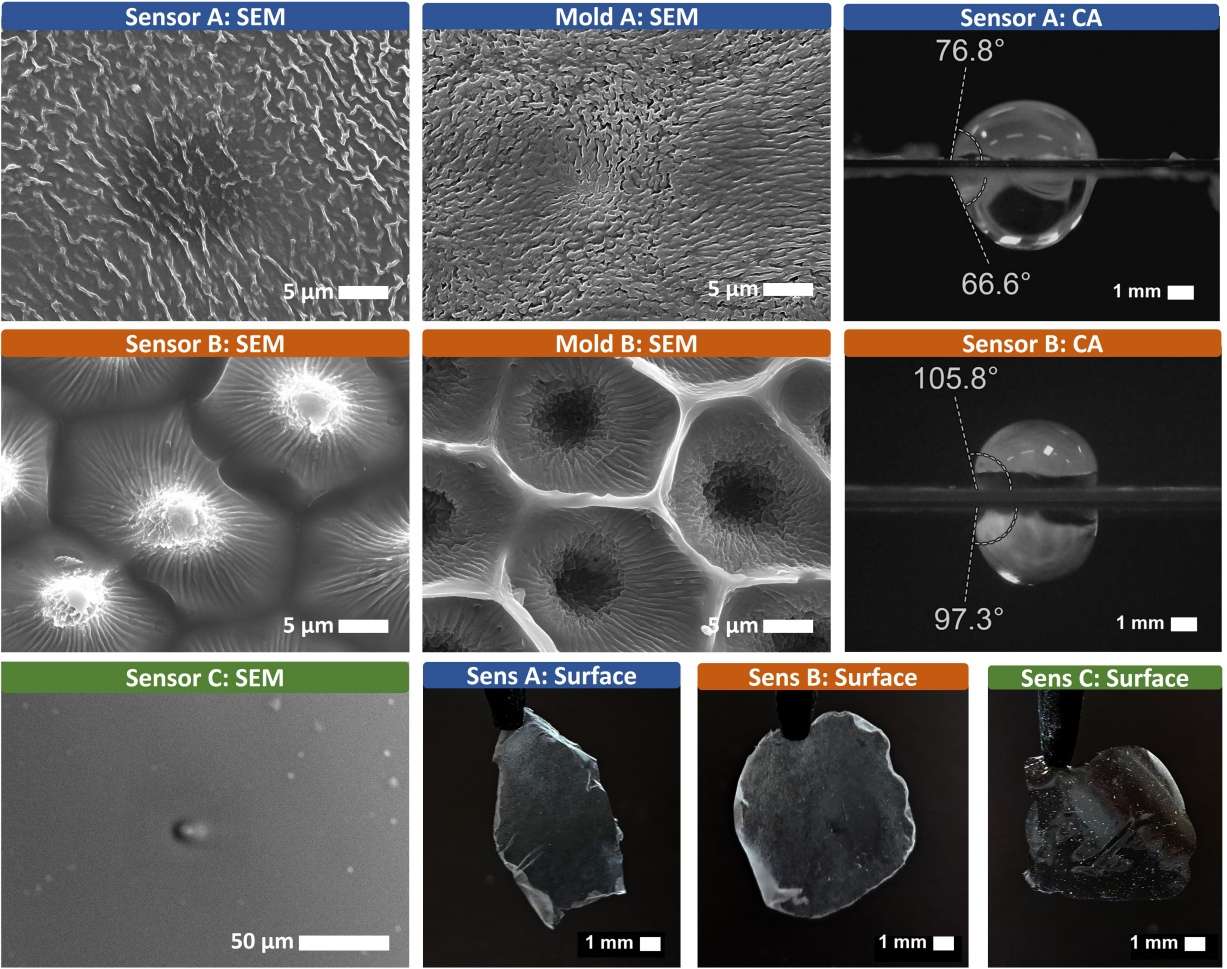
Characterization of sensor surface variations. Surface A replicates PDMS mold wrinkles, enhancing wettability with a CA of 76.8°. Surface B partially reproduces spike patterns, lacking full 3-starred shapes but increasing the CA to 105.8°. Surface C shows minimal roughness with minor imperfections. Light reflection highlights surface microstructure: Surface C appears more transparent, while textured surfaces A and B are more opaque, as shown in the bottom-right images.

**Fig. 3.**
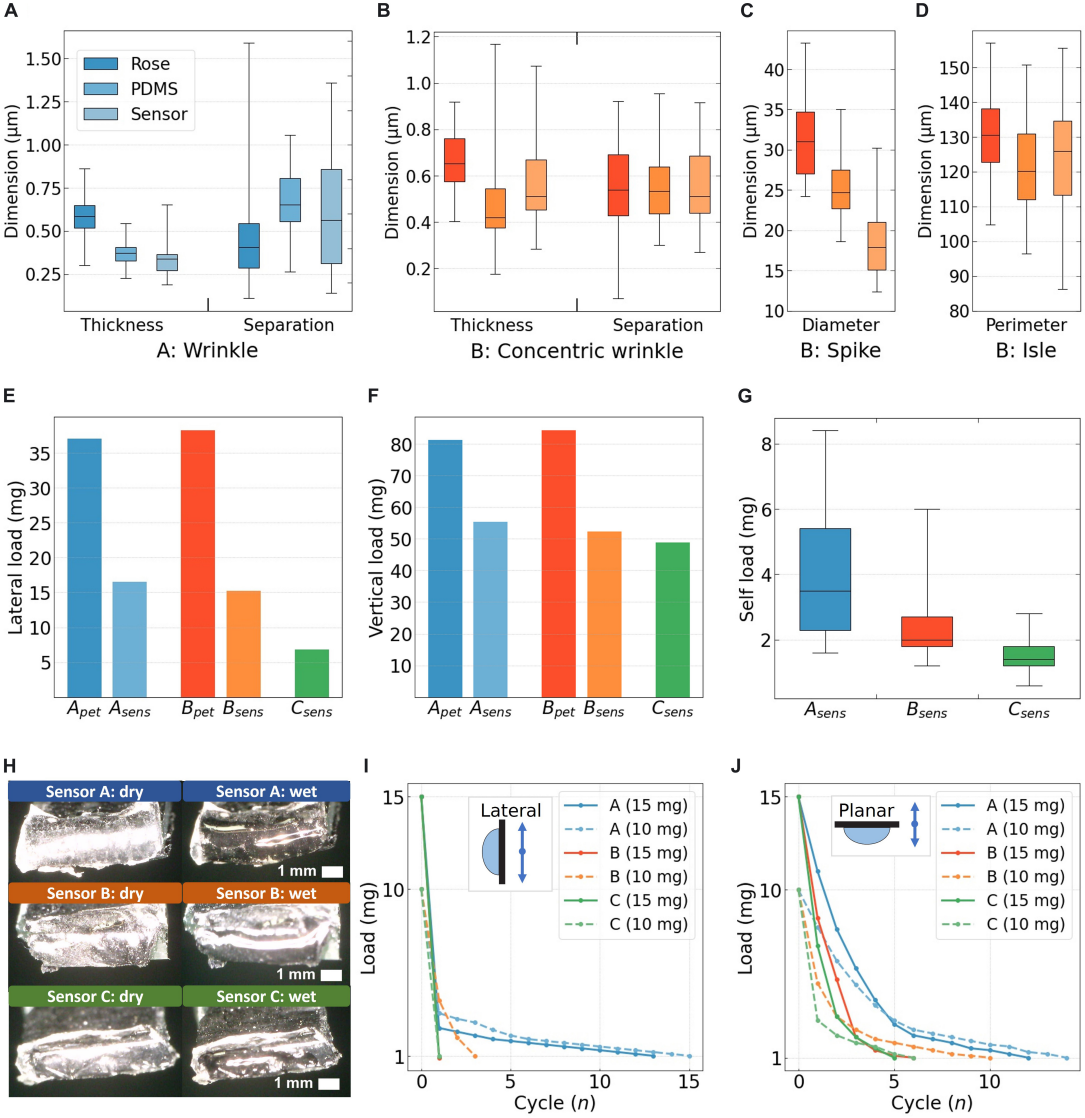
Copy accuracy of rose petal microstructures and water adhesion properties. (A) Type A surface: Wrinkle thickness decreases during replication, while spacing variability increases due to PDMS interaction with delicate rose structures (*n* = 200). (B) Type B concentric wrinkles: Wrinkles thin, but spacing remains consistent (*n* = 200). (C) Type B spikes: Spike diameter reduces by 40% (*n* = 200). (D) Type B isles: Isle perimeters slightly reduce, showing cumulative dimensional instability from PDMS and ISM limitations (*n* = 200). (E) Maximum load perpendicular: Rose petals support heavier loads on a 90° incline than artificial copies, but modified sensors outperform the baseline by >100% (*n* = 50). (F) Maximum load planar: Modified sensors improve load bearing by 5% and 3% over baseline (*n* = 50). (G) Sensor self-adhesion: Sensor A shows superior sweat adhesion compared to sensors B and C. (H) Self-adhered sweat: Surface A demonstrates full wetting, visible through light reflection. (I) Lateral motion cycles: Under 15- to 20-mg loads, sensor A retains >1 mg of sweat for 4+ cycles, outperforming the baseline. (J) Planar motion cycles: Sensor A excels in water retention during repeated cycles.

We obtained the negative structure of the rose petal in order to replicate it on the ISM surface. The microarchitecture of the rose petal was replicated on a PDMS substrate and subsequently on the ISM membrane. SEM images, shown in Fig. 2, depict the negative imprint of the rose surface and the positive replication of the texture on the ISM. Although the replication accuracy of PDMS casting is favorable, submicrometer features tend to become rounded and less detailed. The limitations in mimicking submicrometer structures cause an alteration in water adhesion and discern from their natural counterpart, as listed in Table 2. The partial reproduction of the rose petal effect arises from the aggressive interaction of PDMS on living cells [45]. Comparative analysis was conducted on the topographies of the natural rose petal and the biomimetic surface. The features are defined in Supplementary Information S2, and results are presented in Fig. 3A to D.

**Table 2.** Static adhesion properties

*n* = 50	Petal A	Petal B	Surface A	Surface B	Surface C (*N*)
Maximum lateral load/mg	37.1	38.3	16.5	15.2	6.8
Maximum planar load/mg	81.3	84.4	55.4	52.2	48.9
Self-adhesion/mg	9.1	7.2	3.9	2.4	1.3
Self-adhesion CA/(°)	75	62.1	46.6	58.7	54
Longest meniscus/mm	-	-	46.6	58.7	54

**Fig. 4.**
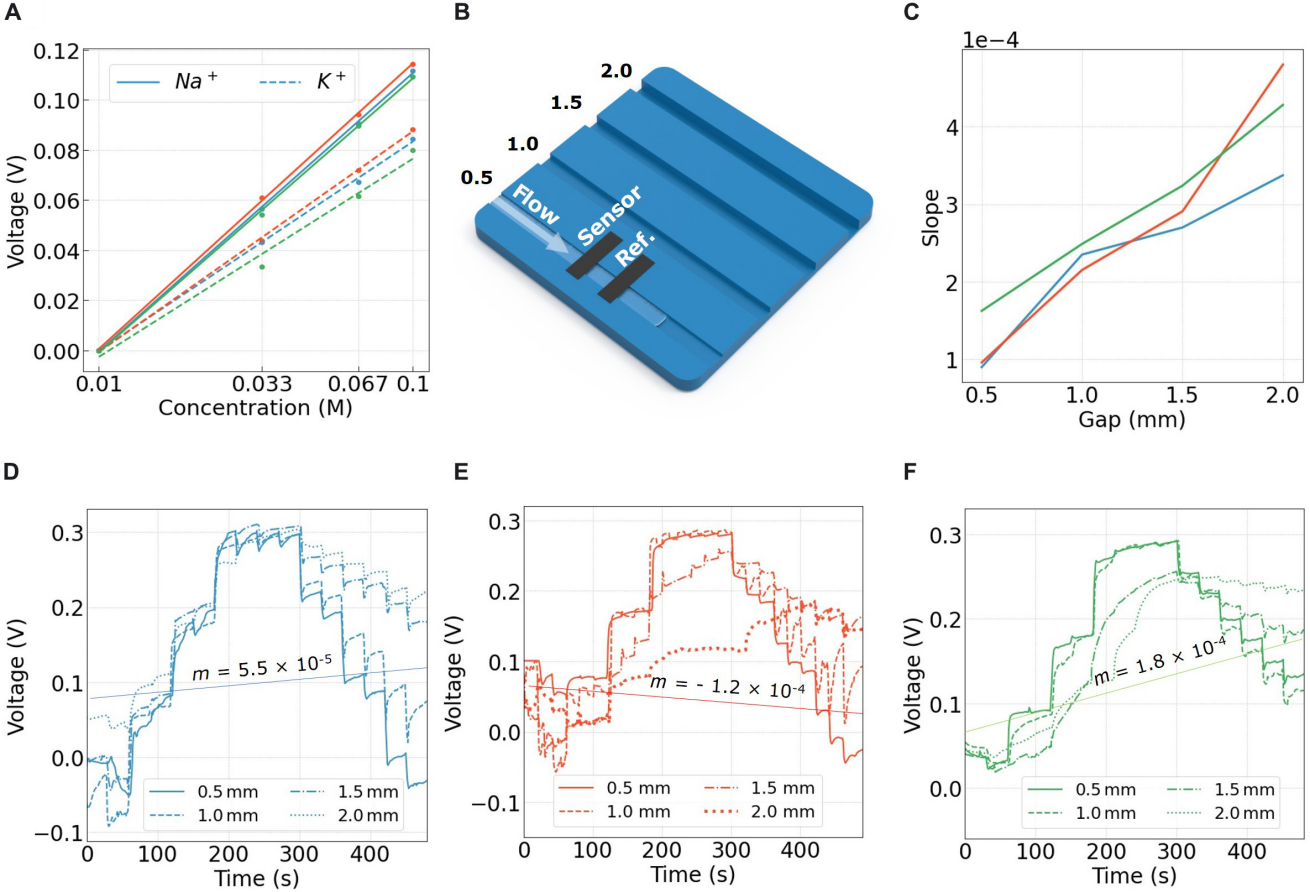
Electrochemical characterization and self-cleaning. (A) Average signal for NaCl and KCl at concentrations ranging from 0.01 to 0.1 M (*n* = 15). Sensor B (red) shows higher sensitivity than A (blue) and C (green), although the signal of the undesired ion proportionally increases, indicating that selectivity cannot be enhanced by the microtexture. (B) 3D-printed structure with channels set to different heights. Sensors are placed on the top of the channel to simulate noncontact measurement. (C) Self-cleaning effectivity at varying channel gaps (*n* = 15). Average slope of the linear regression for the signal indicates the self-cleaning property. Signal drift can be appreciated by ion buildup; an ideal slope would be close to 0. (D to F) NaCl solutions were introduced in ascending and descending concentrations to evaluate the sensors’ reproducibility and ion buildup. Reproducibility was quantified by calculating the slope of the linear regression, with the slope of the 0.5-mm channel provided as a reference. An ideal slope is near 0, indicating minimal drift. Sensor A demonstrated superior performance in terms of self-cleaning and reproducibility compared to other implementations.

The characterization of the reproduced rose surface features is essential to understand the ISM new wetting properties. The natural rose petal morphology, surface type A, is characterized by an organically dispersed pattern of microwrinkles with variable heights (Fig. 2). These wrinkles are approximately 1 μm in width, with embossing features and spacing less than 1 μm apart. The distribution of the heigh difference is in the form of rounded areas, where lower heights comprise the center of the circle and higher heights populate the surrounding of the area. The resulting structure in sensor A partially replicates these features, maintaining a submicrometer wrinkle width and approximately 1-μm spacing, achieving a balanced dimensionality between them. However, the reproduction of lower-height wrinkles is less successful. Despite not fully capturing the rose petal effect, the bioinspired ISM membrane showed an enhancement in wettability, reducing the CA from 90° to 76.8°. The replication accuracy for sensor A is shown in Fig. 3A. The structure of the natural rose petal surface B is composed of polygonal isles containing spike-like protrusions in the center, emerging from 3- or 4-pointed bases. Surrounding each spike are well-defined wrinkles radiating toward the island’s perimeter, the perimeter of the isle, which is slightly raised. This can be observed in detail in Supplementary Information S2. The biomimetically inspired alteration of sensor B resulted in more rounded features and the obscuration of the star-shaped bases. The embossed perimeter has transformed into a beveled edge, effectively inverting the original profile. These modifications may be attributable to PDMS shrinkage during the curing process, [45], or the potential toxicity of PDMS toward cells, as suggested by Roch et al. [46], which could indicate inflammatory responses following polymer implantation. The replication accuracy for sensor B can be observed in graphics in Fig. 4B to D. The transferred ISM achieved a wider CA modifying its original value from 90° to 97.3°, demonstrating enhanced similarity to the petal’s surface properties. The typical resolution achievable with standard PDMS molding is in the range of 1 to 2 μm, while advanced techniques have demonstrated replication down to 20 to 50 nm [47]. In contrast, the limiting factor in microstructure replication lies with the ISM structural material, PVC. Although PVC is an excellent substrate for ionophore integration [48], it is not suitable for replicating fine microstructures, with a theoretical resolution limit of approximately 5 to 10 μm [49].

**Table 3.** Dynamic adhesion properties

*n* = 100	Sensor A		Sensor B		Sensor C (N)	
Initial water load/μl	15	10	15	10	15	10
Planar setup						
Loss after 1st cycle/μl	4	2	7	5	8	7
Maximum cycles to <1 μl (*n*)	12	15	6	11	5	6
Lateral setup						
Loss after 1st cycle/μl	12.5	7	15	6	15	9
Maximum cycles to <1 μl (*n*)	12	14	1	4	1	2

A comparative analysis of droplets on upward- and downward-facing surfaces revealed a close relative difference in CA values for both the rose petal and sensor B, a phenomenon attributed to stronger boundary adhesion countering gravitational forces, consistent with the Wenzel effect [50]. These findings validate the effectiveness of the bioinspired microtexturing, as the enhanced boundary adhesion facilitates droplet retention within the cavities, ensuring optimal sweat interfacing. This improvement supports more reliable electrolyte measurement during sweat analysis. Detailed measurements for surfaces A and B are presented in Tables 4 and 5.

**Table 4.** Side A morphology dimensions

*n* = 100	Rose A			PDMS A			Sensor A		
Wrinkle dimensions	Min	Max	Avg	Min	Max	Avg	Min	Max	Avg
Thickness/μm	0.30	0.86	0.58	0.23	0.55	0.37	0.19	0.65	0.34
Separation/μm	0.30	1.6	0.46	0.27	1.1	0.66	0.14	1.4	0.60
Low area perimeter/μm	40	140	84	57	183	103	38	110	75

**Table 5.** Side B morphology dimensions

*n* = 100	Rose A			PDMS A			Sensor A		
	Min	Max	Avg	Min	Max	Avg	Min	Max	Avg
Spike dimensions									
Base diameter/μm	24	43	31	19	35	25	12	30	19
Wrinkle thickness/μm	0.32	0.83	0.52						
Wrinkle separation/μm	0.12	0.72	0.31						
Star points (*n*)	3.0	4.0	3.2						
Island dimensions									
Base perimeter/μm	104	157	130	96	151	121	86	156	124
Wrinkle thickness/μm	0.40	0.92	0.66	0.18	1.2	0.50	0.28	1.1	0.56
Wrinkle separation/μm	0.07	0.92	0.66	0.30	0.96	0.55	0.27	0.92	0.55
Geometry sides (*n*)	5	7	5.9	5	7	6.0	5	7	5.95

### Hydrodynamic properties

To evaluate the sensors’ performance in retaining water, we first determined the maximum static load capacities for each surface variation in static conditions. For clarity, the untreated ISM is referred to as sensor C. Bioinspired sensors A and B demonstrated marked improved static water retention compared to sensor C. The naturally occurring microstructure of the rose petal still surpassed the bioinspired ISM in static load capacity, both face-down and laterally. Detailed results are presented in Fig. 3E and F, highlighting the comparative performance of the sensors against the natural rose petal benchmark.

Achieving low-contact measurement relies on the sensor’s ability to retain liquid through intrinsic forces. Self-adhesion is defined as the ability of a surface to lift water by tension generated at the interface. Among the ISM variations, sensor A exhibited the highest self-adhesion capacity, with an average increase of 200% compared to the baseline sensor C. Sensor B demonstrated a more modest improvement, achieving an 84% increase relative to sensor C. The results are visualized in Fig. 3G, showing the comparative performance across sensor variations. The impact of self-adhesion is also evident in the visual comparison of sensors before and after contact with water, evident through changes in light reflection, as shown in Fig. 3H.

Another factor influencing the stability of the readouts is the capacity of the sensor to retain water under dynamic forces. An experimental setup was designed to account for normal and parallel forces relative to the sensor plane to evaluate adhesion performance comprehensively. A custom 3D-printed structure was employed to ensure precise control and reproducibility of the movements. Normal forces were applied in the planar configuration, with the water droplet positioned face-down on the sensor surface. Parallel forces were tested in a lateral configuration, where the water droplet adhered to a 90° oriented sensor surface, simulating the gravity and shear effects. A schematic representation of the applied forces and their orientations is provided in Fig. 3I and J. This setup enabled a detailed assessment of the sensor’s water adhesion properties under varying mechanical stress directions. The results of the lateral motion experiment revealed that surface A exhibited the highest lateral water adhesion among the tested surfaces, outperforming both surfaces B and C by enduring a greater number of motion cycles (Fig. 3J). This enhanced performance is attributed to the wrinkles reproduced from the back side of the rose petal (surface A) and transferred to the sensor. Bioinspired microstructures demonstrated an exceptional ability to retain water during lateral motion, surpassing other surface types. This test was also used to characterize the self-cleaning effect. When a surface loses most of its water under one cycle, we consider this surface to be self-cleaning. This effect is influenced by the initial load placed on the surface. Two dynamic tests were conducted using applied loads of 10 and 15 mg. It was observed that at 15 mg, the load exceeded the threshold associated with the rose petal self-cleaning effect, thereby triggering the self-cleaning mechanism and losing all the liquid load within one cycle. Therefore, optimizing the balance of the self-cleaning properties of the microstructured surface is crucial to ensure reliable signal acquisition. The sensor must retain fluid effectively during periods of low flow while allowing efficient release during high flow conditions, thereby maintaining stable operation across varying sweat production rates (Table 3).

### Electrochemical sensing

**Fig. 5.**
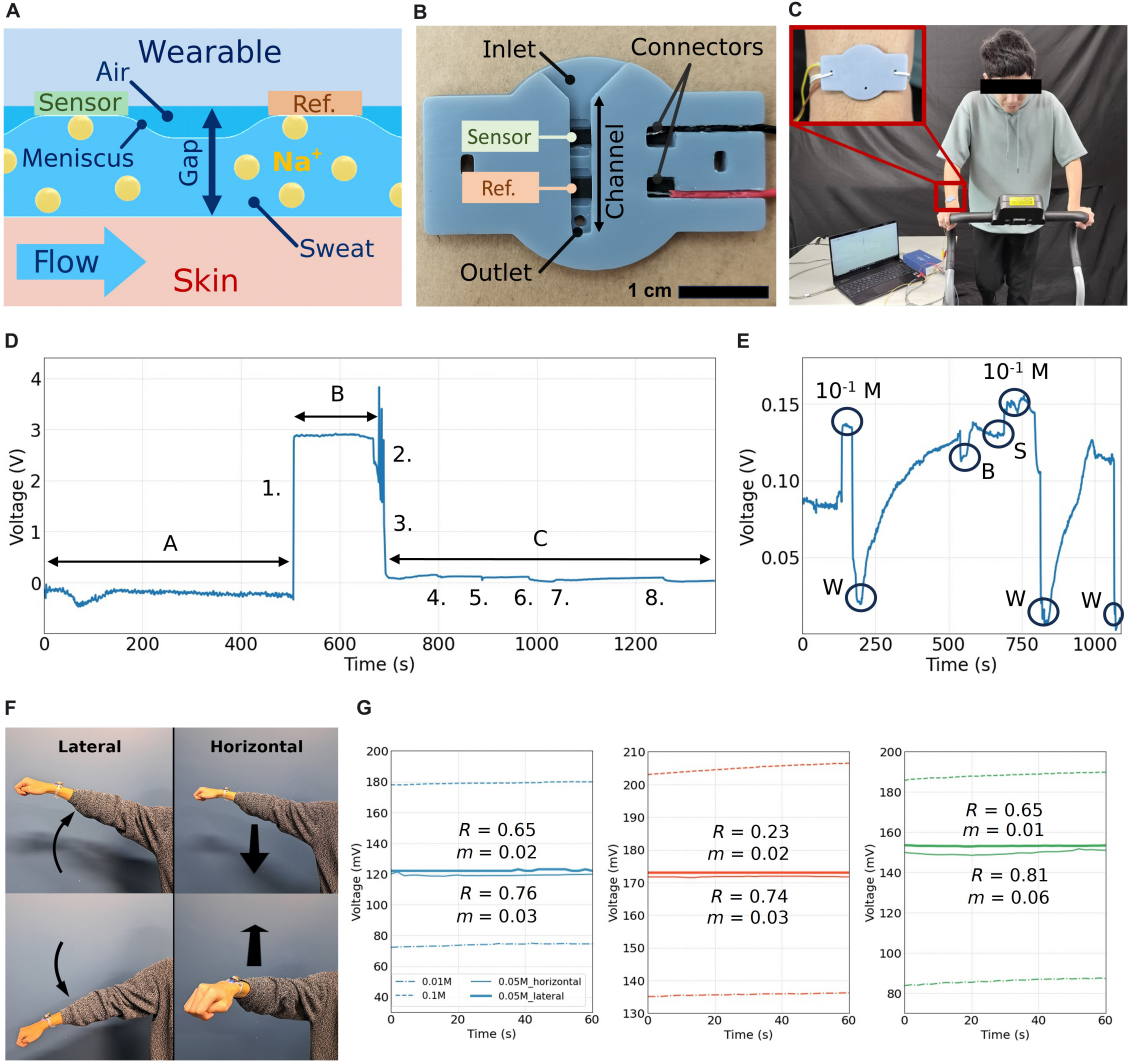
Wearable device and real-time on-body testing. (A) Cross-section of the sweat channel showing meniscus formation. Electrodes remain in the same medium due to water tension despite air gaps. (B) 3D-printed wearable device with a funnel-shaped inlet for sweat collection and a sealed outlet to prevent leaks and noise. (C) Running experiment setup: The device is secured on the wrist with rubber bands and connected to a potentiostat for OCP measurement during treadmill running. (D) Sodium ion monitoring during a 20-min session showing phases: (A) air noise, (B) reference electrode wetting, and (C) real-time measurement. Key events include sweat contact (1), channel filling (3), bubble effects (5), and sample injection for recirculation testing (6, 8). (E) Sweat recirculation testing reveals signal stability depending on sweat production. Bubble disturbances (B) are brief, and external access allows recalibration using controlled sodium in water solutions (M) and DI water (W), demonstrating rapid response and repeatability when sweat is produced (S). (F) Use case scenario demonstrating lateral and horizontal movements. The sensor was integrated into a wireless device to provide greater freedom of movement during testing. (G) Signal stability during lateral and horizontal movements. The signal remained unaffected by dynamic forces, even when using the deepest sensing channel of 2 mm. The measured signal consistently stayed within the calibration thresholds, ensuring reliable performance under dynamic conditions.

Sensitivity and selectivity were characterized to assess the effects of modifying the ISM surface. The modified bioinspired ISM offered higher sensitivity than the baseline. The sensor’s responsiveness was evaluated using NaCl and KCl solutions at varying concentrations, from 0 to 10^−1^ M. Sodium ion concentration in human sweat was found to be less than 10^−1^ M, as seen in Fig. 5E. The results from the measurement can be found in Fig. 4A and Supplementary Information S3. The theoretical electrolyte sensitivity can be calculated using the Nikolskii–Eisenman equation:

$$E=\text{const}.+\frac{2.303RT}{zF}\text{log}C$$ (1)

where *R* is the gas constant, *T* is the temperature in K, *F* is the Faraday constant, and *z* is the ion charge (Na^+^ = 1) [51]. The ISM for surface A has a sensitivity of 76.21% relative to its theoretical value, while surfaces B and C exhibit sensitivities of 82.43% and 74.21%, respectively. Data detailing sensitivity and selectivity experiment results can be found in Supplementary Information S4 and S5. We concluded that the improved sensitivity could be attributed to the increased interfacing area provided by the bioinspired 3D structures, as no other inherent factors could account for this change. These structures introduce new contact regions compared to the baseline, substantially enhancing ion exchange and transport efficiency. Since all ISMs were prepared from the same cocktail batch with homogeneously dispersed ionophores, the observed sensitivity enhancement is solely linked to the structural modifications. The introduction of 3D structures also leads to variations in membrane thickness compared to the baseline. However, the impact of increased surface area significantly outweighs that of thickness changes in enhancing sensitivity [52]. The incorporation of ripples and microstructures within the ISMs boosts the probability of ionophores interacting with and transporting ions, thereby improving overall sensor performance. The surface increment for sensor A is calculated based on the amount of surface covered by ripples. The added surface equates to a curved surface of the area of a half cylinder. The increment can be calculated as follows:

$$\text{Area Increment Ripples}\;=\frac{\text{Tflat}+\left(\sum \text{Acurved}-\sum \text{Aflat}\right)}{\text{Tflat}}\times 100\%$$ (2)

where Tflat = *H*·*W* total area of the image, Aflat = *L*·*D* is the equivalent area of the wrinkle, and Acurved = π*r*·*L* is the new area added. *H* is the height of the picture, *W* is the width of the picture, *L* is the wrinkle length, *D* is the diameter of the wrinkle, and *r* is the radius of the wrinkle. We used ImageJ to calculate the total length of a ripple in an 8 μm × 6 μm area, resulting in a total of 20 μm length. Wrinkles were measured at an approximate diameter of 0.35 μm. Using the formula, the resulting increment was a 16% increase in contact area.

For surface B, the area increase can be calculated by the number of peaks and the average cone height and diameter.

$$\text{Area Increment Spikes}=\frac{\text{Tflat}+N\cdot \left(\text{Acone side}-\text{Abase}\right)}{\text{Tflat}}\times 100\%$$ (3)

where $\text{Acone side}=\sqrt{r2+h2}$, $\text{Abase}=\pi r^{2},$ and *N* is the number of peaks of complete cones within the picture. *R* is the radius of the cone, and *h* is the height of the cone. We calculated the area increment in a surface of 80 μm × 60 μm area with 5 peaks of radius 18 μm and height 32 μm averages, resulting in an increment of 22% of the surface area.

The theoretical surface area increase closely aligns with the sensor characterization results shown in Fig. 4A, suggesting that the observed improvement in sensitivity may be attributed, at least in part, to the enhanced contact area introduced by the surface texturing.

Noncontact data acquisition is a feature of the enhanced ISM, allowing sweat to be analyzed at a distance from the exudation area. To characterize noncontact signal acquisition, liquid solutions were flown at varying air gaps from the sensor utilizing a 3D-printed channel as illustrated in Fig. 4B. Sample collection relies on the sensor’s adhesion mechanism and enables real-time measurements as demonstrated with the rapid response to changes in ion concentration flown through the channels (Fig. 4D to F). Samples with varying NaCl concentrations (0 to 10^−1^ M) were introduced in ascending and descending orders at fixed intervals to evaluate the sensors’ response times. Stabilization times were observed on the scale of tenths of seconds. The channel gap influenced ion removal, particularly during the descending phase of the experiment. As shown in Fig. 4D to F, flushing the channels with DI water did not restore the signal to its initial baseline value, indicating incomplete ion rinsing. Self-cleaning behavior was quantified by the slope of the linear regression applied to the signal (Fig. 4D to F). A steeper slope indicated a loss of self-cleaning efficacy, leading to signal drift caused by residual ion accumulation on the sensor. An optimal slope, close to zero, would reflect minimal drift and effective self-cleaning. While sensor B exhibited the flattest slope for channels up to 1 mm, its performance declined in taller channels, indicating its inability to maintain stability under these conditions. In contrast, sensor A demonstrated a consistent linear decline in performance, starting from a level comparable to sensor B in smaller channels. Sensor C, serving as the baseline, was outperformed by the bioinspired sensors.

Meniscus formation, driven by surface tension at the liquid interface, also impacted measurement effectiveness during flow. This phenomenon, illustrated in Fig. 5A, contributed to a dampening effect during descending flow, where the syringe pump’s back-and-forth motion caused ions to be intermittently removed (signal decreases) and reintroduced (signal increases), as seen in Fig. 4D. This dampening effect was less pronounced in sensors B and C (Fig. 4E and F), correlating with their reduced ability to retain liquid at larger channel gaps, likely due to weaker surface tension forces.

The bioinspired membrane based on surface A of the rose petal effectively addressed adhesion and self-cleaning challenges. By promoting consistent liquid retention and reducing the formation of air gaps, sensor A demonstrated superior performance, maintaining reliable readouts under dynamic conditions. It exhibited no failed readouts, even at the widest gap setting of 2 mm, and consistently demonstrated the best self-cleaning characteristics among the tested sensors.

### Running experiment

The bioinspired sensor was embedded in a wearable device and tested under real-world conditions during a high-intensity exercise session to assess sweat adhesion and recirculation. A 3D-printed hard resin wearable was employed to securely position the sensor on the skin, featuring a sensing channel with a 2-mm gap. This gap was chosen to avoid channel blockage caused by the skin’s flexibility and morphological variations, as well as to eliminate the need for nonreusable solutions like tape or glue commonly reported in previous studies [27–32]. Flexible embodiments, such as PDMS structures and flexible UV resin, failed to establish a tight seal between the skin and the sensing channel. Flexible channels did not provide stable signals, as sensor deformation introduced additional noise, compromising signal quality. At its current stage of development, the CNTF sponge lacks the mechanical durability to endure the repeated deformation cycles required for use in flexible devices during sporting activities. However, we anticipate that ongoing advancements in the fabrication of highly sensitive and mechanically robust sponge-based composite electrodes will eventually overcome this engineering limitation [53]. These design iterations are discussed in detail in Supplementary Information S6 to S8.

An overview of the rigid wearable design, highlighting the sensing channel, is presented in Fig. 5B. The wearable was positioned on the dorsal wrist, identified as the optimal site for sweat collection (Fig. 5C), consistent with findings by Kim et al. [54]. Alternative locations, including the underarm, upper arm, and areas near the armpit, were also evaluated. However, the dorsal wrist demonstrated superior performance, offering the highest sweat volume due to its anatomical position as an endpoint of the arm, coupled with enhanced wearing comfort during extended use.

During the 20-min exercise session, the sensor’s response to electrolyte concentration was monitored (Fig. 5D). Initially, the sensor channel contained air, the electrochemical readout circuit remained open, and noise was present in the signal. After 8 min, sweat began to enter the channel, initially adhering to the reference electrode. By minute 11, the channel started to fill, and the partial filling of the channel appears as intermittent spikes in the signal. Once it was completely filled, the signal stabilized, marking the start of real-time measurement. Several key observations were made: The signal increased progressively as sweat initially carried ions from the skin. A sudden downtrend spike indicated bubble formation in the channel. The arm was actively wetted with a low-concentration 10^−3^ M NaCl solution to test sweat recirculation and demonstrate sensor recalibration. The channel was then filled with sweat after 25 s. The arm was wetted again with the low-concentration solution, and the signal remained low, indicating reduced sweat production and prolonged liquid retention in the channel. This demonstrated the sensor’s adhesion capability, preventing the channel from emptying completely during periods of low sweat production. Another remarkable point is the downtrend of the signal stabilization. As the experiment progresses, sodium concentration signal stabilizes at lower values, indicating the loss of electrolytes in sweat. The exercise can be viewed in Movie S1.

The recirculation process is a critical aspect of this study, showcasing the potential continuous operation of the device and its improved comfort due to the nonstagnant sweat collection as compared to other studies where water adhesion is used to take advantage of capillary flow in exchange of sweat recirculation [55,56]. When the arm was actively wetted with varying sodium concentrations, the sensor demonstrated response times within seconds, with gradual signal stabilization (Fig. 5E). However, accurately determining sodium concentration from freshly produced sweat depends heavily on the user capacity to fill the sensing channel and the capacity to keep sensors calibrated during use; slower sweat production can take minutes to yield precise readings, and a recirculation capacity is necessary to reflow the sensing channels with calibration samples. The self-cleaning mechanism of the rose-inspired sensor played a critical role in addressing irregular sweat flow. In this design, sweat recirculation is governed by an overflow-based mechanism: During periods of low sweat production, the sensor retains fluid within the channel, and once a defined threshold is exceeded—determined by the maximum tolerable lateral and planar loads—the self-cleaning process is activated. This mechanism ensures signal stability and prevents the formation of bubbles that could lead to sudden spikes in the signal.

Additional movements were evaluated using a wearable device powered by a battery and equipped with wireless communication, enabling the characterization of a wider range of motions. Two additional movements were tested to assess signal stability as seen in Fig. 5F. First, the sensor variations were calibrated, and upper and lower thresholds were established. Subsequently, a sample with a concentration falling between these thresholds was measured. The results demonstrated that sensors retained liquid within the channel, even at a depth of 2 mm, during dynamic movements as seen in Fig. 5G and H. We stablished slope and linear regression as comparison parameters. All sensor variations demonstrated no movement-induced effects on the signal, as confirmed by fast Fourier transform (FFT) analysis, which showed no peak at the arm oscillation frequency (~5-s band). Due to the wearable device’s 2-s sampling rate, movement had to be kept slow to ensure accurate data acquisition. Drift and instability remained consistent with static experiments as seen in the slope value, indicating minimal motion artifacts. However, differences in electrode–liquid contact were observed over time, with sensor A maintaining stable connectivity for a longer duration in both horizontal and vertical orientations compared to sensors B and C. This behavior aligns with the experimental findings presented in Fig. 3I and J, further supporting the enhanced adhesion properties of sensor A.

## Conclusion

Contrary to the prevailing trend in wearable sweat sensing, which often relies on narrow microfluidic channels or absorbent patches designed to collect minimal volumes of sweat directly from the skin, we intentionally adopted a channel design with a wider 2-mm gap. This design choice was motivated by the need to increase user comfort, reduce skin irritation, and avoid the use of adhesives or conformal contact—features that are particularly important for long-term or high-mobility applications.

While this wider-gap, noncontact approach offers distinct ergonomic advantages and aligns with our goal of creating a more comfortable and reusable sensor, it introduces a unique set of engineering challenges. These include ensuring consistent fluid retention, maintaining reliable sensor-to-liquid contact, and preserving measurement stability despite variable sweat production and movement artifacts. Achieving these goals required us to develop novel strategies for fluid recirculation and self-cleaning mechanisms, which are not commonly addressed in conventional sweat sensor designs. An example of the system’s integration into a low-power, battery-driven device is provided in Supplementary Information S9. The resulting device is comparable to recent academic advancements that emphasize industrial applicability while also addressing the added challenge of enabling noncontact measurement and providing an alternative to no-contact sensors for health monitoring such as volatile organic compound sensors and optical sensors.

The enhanced sweat adhesion and noncontact sensing capabilities of the proposed bioinspired sensor make it particularly well-suited for integration into prosthetic limbs, wearable exoskeletons, and other human–machine interface. Noncontact sensors minimize user fatigue and enable long-time continuous monitoring [57,58]. In these applications, continuous monitoring of electrolytes such as sodium (Na^+^) could serve as an early indicator of dehydration, muscle fatigue, or thermal stress—factors that directly affect user comfort, safety, and device performance. For instance, integrating this sensor into a prosthetic socket could help detect fatigue, prompting adaptive changes in pressure or ventilation. In exoskeletons used for rehabilitation or industrial support, real-time sweat analysis could inform intelligent feedback systems to optimize user effort and prevent overexertion. These examples highlight the translational potential of our design beyond traditional wearable health monitoring.

Advancements in microtexture mold production for replicating 3D structures are expected to improve replicating mechanisms and refine sweat adhesion properties. Less invasive replication methods are to be developed to lessen the strain on biological samples, which cause partial replication of submicrometer scale details. Our current replication method is constrained by the material properties of commercially available substrates such as PVC. Despite these limitations, we were able to partially replicate structural features below the theoretical resolution limit of 5 μm. We acknowledge the inherent challenges of this approach and anticipate that future advancements in polymer chemistry will yield improved materials specifically optimized for high-fidelity texture replication. The methods used to replicate rose petal microstructures may also inspire broader applications. Methods described in this study can be replicated into any PVC-based ionophore sensor such as copper (Cu^2+^) [59], zinc (Zn^2+^) [60], cadmium (Cd^2+^) [61], and lead (Pb^2+^) [62], which can be potential indicators of body ailments such as early Alzheimer’s disease [63] or blood metal poisoning [64].

The findings of this study have the potential to advance the development of thin-film sensors for wearable technologies. By enabling researchers to collect extensive data on body electrochemistry, this work offers new opportunities for in-depth health diagnostics, paving the way for applications in personalized medicine and real-time health monitoring. Furthermore, integrating these sensors with artificial intelligence (AI)-driven computational methods enhances experimental diversity and enables real-time data analysis and predictive modeling. By leveraging machine learning algorithms, the system can identify trends in electrolyte fluctuations, detect anomalies, and predict dehydration risks before symptoms manifest. Such predictive capabilities could be particularly valuable for athletes, individuals in extreme environments, and patients requiring continuous hydration monitoring, making this technology a key innovation in proactive health management.

## Ethical Approval

For this study, a certificate of approval for research with human subjects was approved on 2024 March 7 by the Ethics Review Committee on Research with Human Subjects of Waseda University with Application Number: 2023-498 with the subject Real-time measurement of sweat.

## Data Availability

Source data are provided with this paper.
