# Supplementary material for: Bioinspired Microtexturing for Enhanced Sweat Adhesion in Ion-Selective Membranes
Source: Cyborg Bionic Syst. 2025 Aug 5;6:0337. doi: 10.34133/cbsystems.0337 (PMC12322491; doi:10.34133/cbsystems.0337)
Supplement: Supplementary 1 — Figs. S1 to S9 Movie S1 [file cbsystems.0337.f1.zip › Supplementary Information.docx]

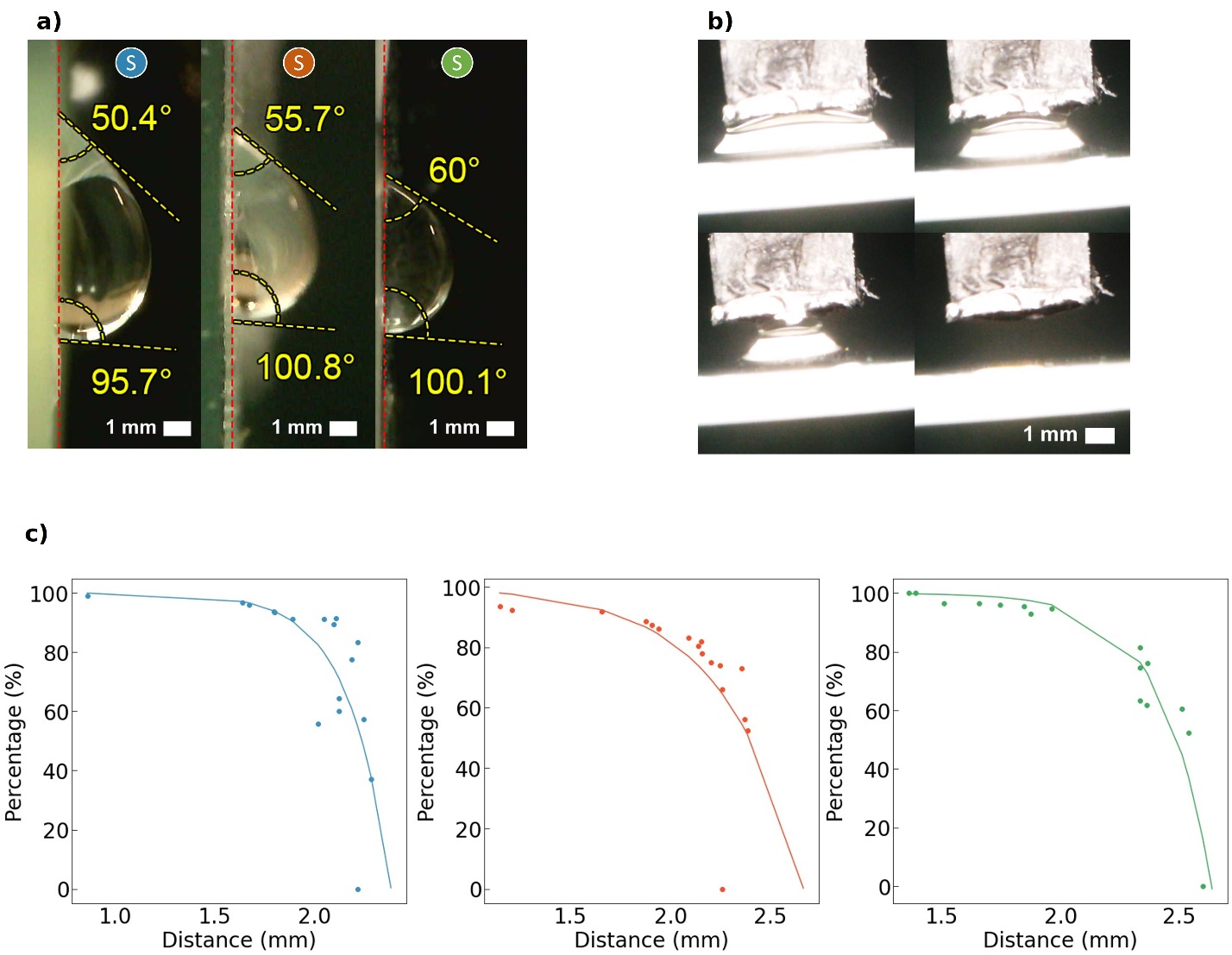


**Supplementary Figure 1 | Contact angle on perpendicular surface and meniscus formation.** (a) The contact angles of the sensors were evaluated during the advancement of the droplet under its own weight. Notably, Sensor C exhibited the smallest lateral load static capacity, resulting in a smaller droplet size. The advanced and receding contact angles were measured to calculate hysteresis, providing insights into the surface tension dynamics of the water droplet and the critical breaking point that causes the droplet to detach.

(b) The progression of the meniscus was observed as the sensor separated from the water body, capturing the dynamic interaction between the sensor surface and the liquid.

(c) The percentage of the sensor surface covered by the meniscus was assessed as the sensor moved away from the water body. All implementations exhibited a similar maximum meniscus extension, with a peak length of 2.5 mm. This value represents the theoretical maximum gap for maintaining a stable meniscus, which served as a reference for designing the 3D-printed tool used to simulate maximum channel depth.

**
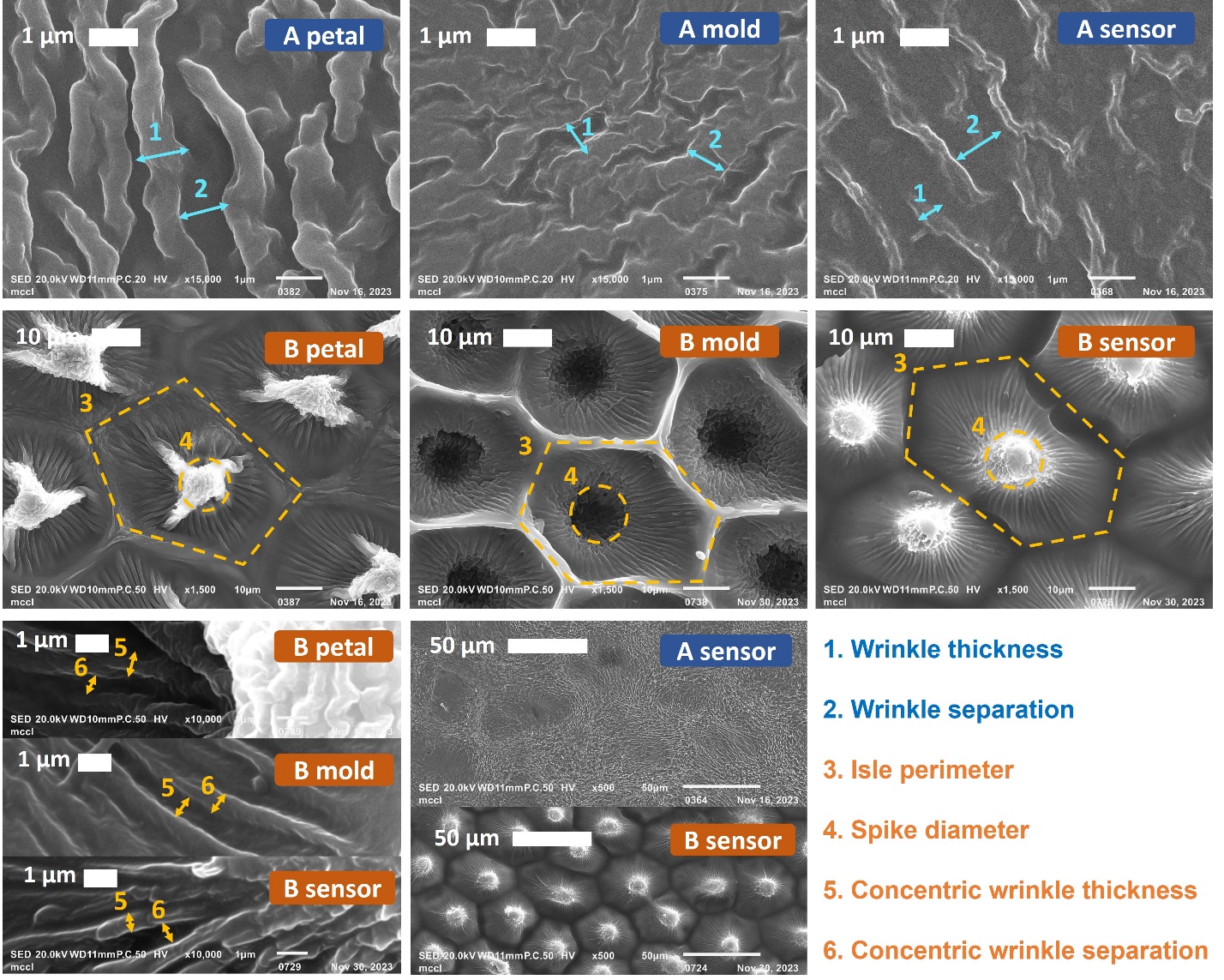
**

**Supplementary Figure 2 | Definition of micro texture features.** The rose petal structures, designated as Sides A and B, exhibit distinct features that are systematically defined and analyzed through the progression of the replication process: from the original biological structure to the negative mold and finally to the ion-selective membrane (ISM). A sequential examination reveals significant shrinkage of the features during replication, attributable to material limitations and the fragility of the organic textures.

The curing process of PDMS introduces shrinkage that compromises the replication of delicate structures, such as the intricate three-point star bases, which are completely lost. However, larger surface areas are replicated with high yield, demonstrating the effectiveness of the process for macro-scale features. Sub-micron structures surrounding Side B are well-preserved, indicating that the copying mechanism itself is robust and that the primary limitation arises from the structural fragility of the biological sample.

At larger scales, the surface replication appears complete and uniform, with no discernible empty areas, validating the reproducibility and suitability of the process for creating functional textured surfaces. Despite some limitations in fine detail replication, the method achieves high fidelity in replicating critical surface features necessary for ISM applications.


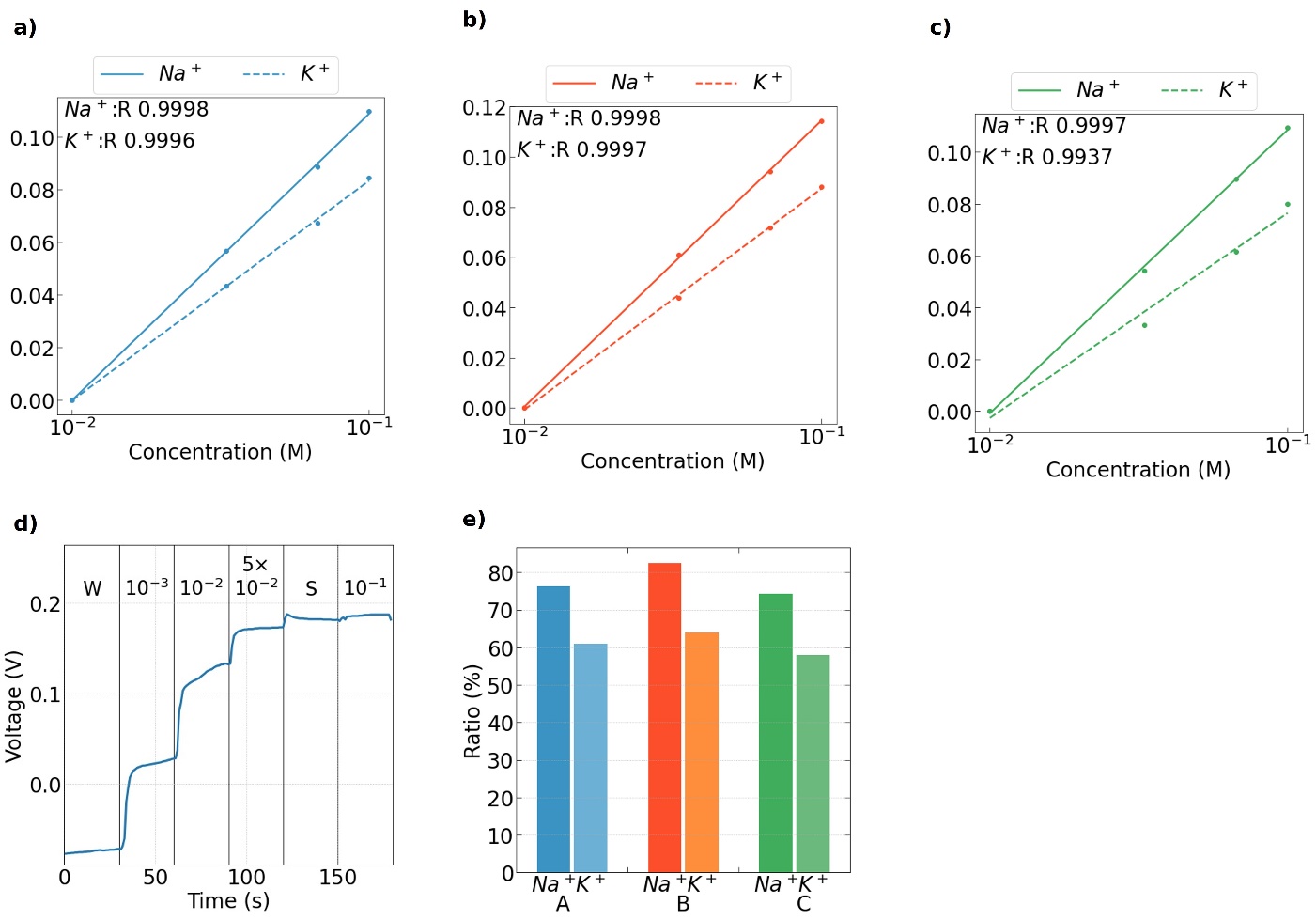


**Supplementary Figure 3 | Ion sensitivity at determined epochs.** The comparison of sodium (Na+) and potassium (K+) ions detected by the ion-selective membrane (ISM) at fixed concentrations ranging from 0 M to 10^-1^ M highlights the sensitivity differences among the tested configurations. Results show that:

- (a) Side A and (b) Side B exhibit higher sensitivity compared to (c) Side C, aligning with their increased interfacing area due to bio-inspired 3D structures.
- While the sensors demonstrate improved sensitivity, selectivity for Na+ over K+ does not show significant enhancement. The bio-inspired modifications appear to increase the sensitivity for both ions proportionally, indicating that selectivity has not been favorably influenced.

(d) Continuous sodium ion measurement during step-by-step concentration changes indicates that Sensor A was the most suitable for real-world application. Sensitivity tests confirmed its capability to detect sodium ion concentrations in natural sweat, typically ranging between 10^-3^ M, 10^-2^ M, 3.3×10^-2^ M, 6.6×10^-2^ M, 10^-1^ M demonstrating compatibility with human physiological levels.

A few observations were noted:

1. **Sensitivity Reduction Over Decades:** Sensitivity diminishes as ion transporters approach saturation due to the exponential nature of charge accumulation.
2. **Stabilization Time:** Signal stabilization is not instantaneous, ranging from seconds to tenths of seconds, depending on the flow dynamics in the channel.
3. **Free-Flow Limitation:** Unlike capillary-flow systems, the free-flow approach relies heavily on sweat volume to fill the channel rapidly, resulting in variability in response times during recirculation events.

These findings confirm that while the bio-inspired ISM sensors effectively detect sweat sodium levels, further optimization in selectivity and stabilization speed could enhance their performance in real-world applications.

(e) Theoretical maximum sensitivity was calculated using the Nikolskii-Eisenman equation. While the incorporation of 3D structures enhanced the sensitivity of the sensors, the selectivity remained unchanged, as it is directly proportional to the sensitivity improvement introduced by the structural modifications.


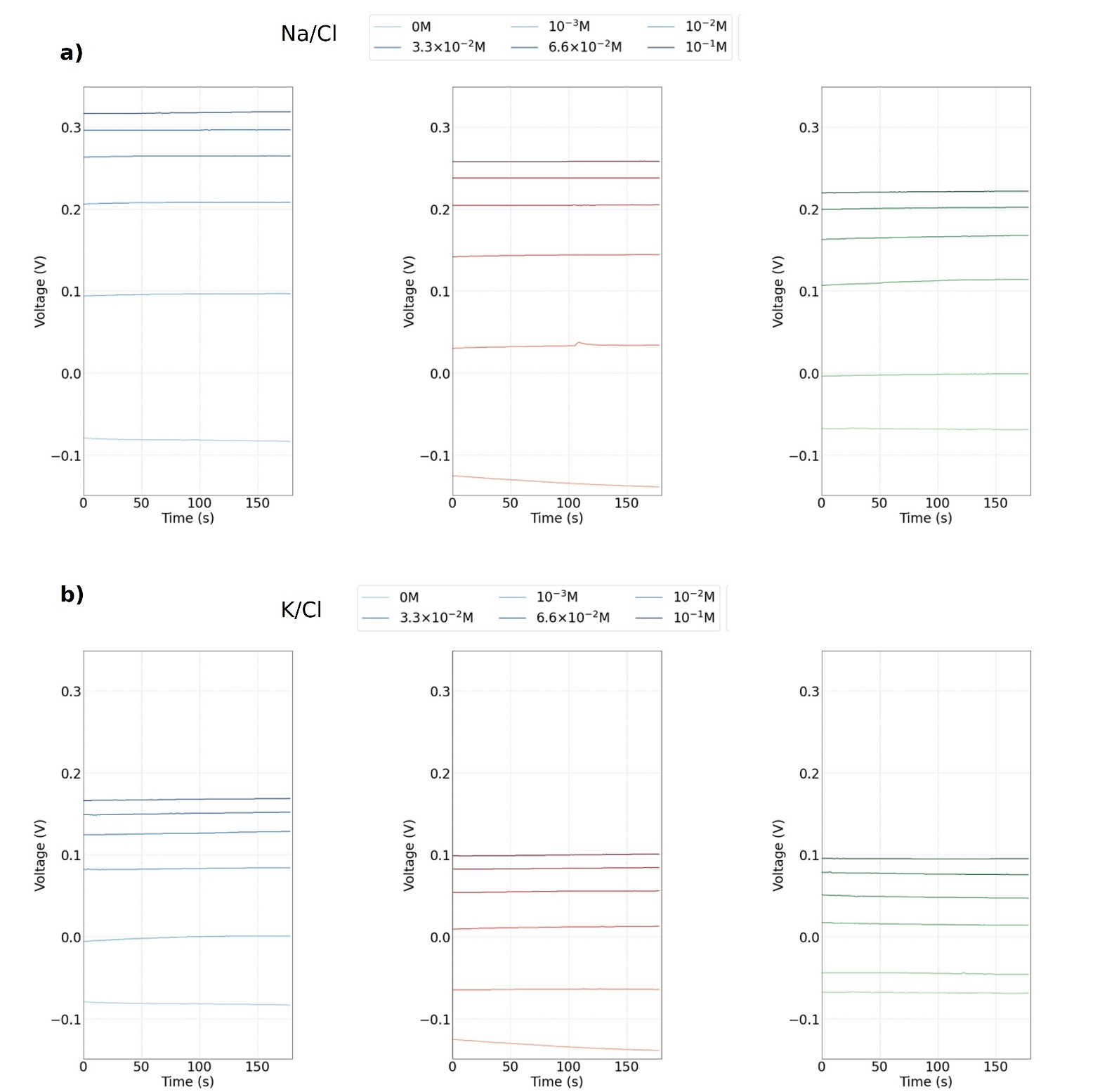


**Supplementary Figure 4 | Stability of the sensor signal.** (a) Signal Stability for Na/Cl: The signal stability for sodium chloride (Na/Cl) dissolved in deionized water was evaluated for Sensors A, B, and C, over a 3-minute interval. Signal drift was observed in all sensors, attributed to the inherent susceptibility of the technology to environmental factors such as temperature fluctuations, humidity, and other uncontrolled extrinsic variables. This underscores the need for a system capable of real-time recalibration, which motivated the development of a recirculation mechanism independent of capillary-flow. The recirculation approach ensures continuous sampling and reduces the impact of transient environmental changes on sensor performance.

(b) Signal Stability for K/Cl: The stability of potassium chloride (K/Cl) signals was also analyzed for Sensors A, B, and C. Sensitivity to potassium ions was consistently lower compared to sodium ions; however, drift was still present across all configurations. To quantify sensor performance, the average signal values were used to calculate the theoretical sensitivity based on the Nikolskii-Eisenman equation. Despite the reduced sensitivity, the persistence of signal drift emphasizes the importance of refining environmental compensation strategies and maintaining calibration for accurate measurements.

These findings highlight the necessity of addressing both drift and recalibration in ISM-based sensor systems, particularly when deployed in dynamic environments for real-time sweat analysis.

**
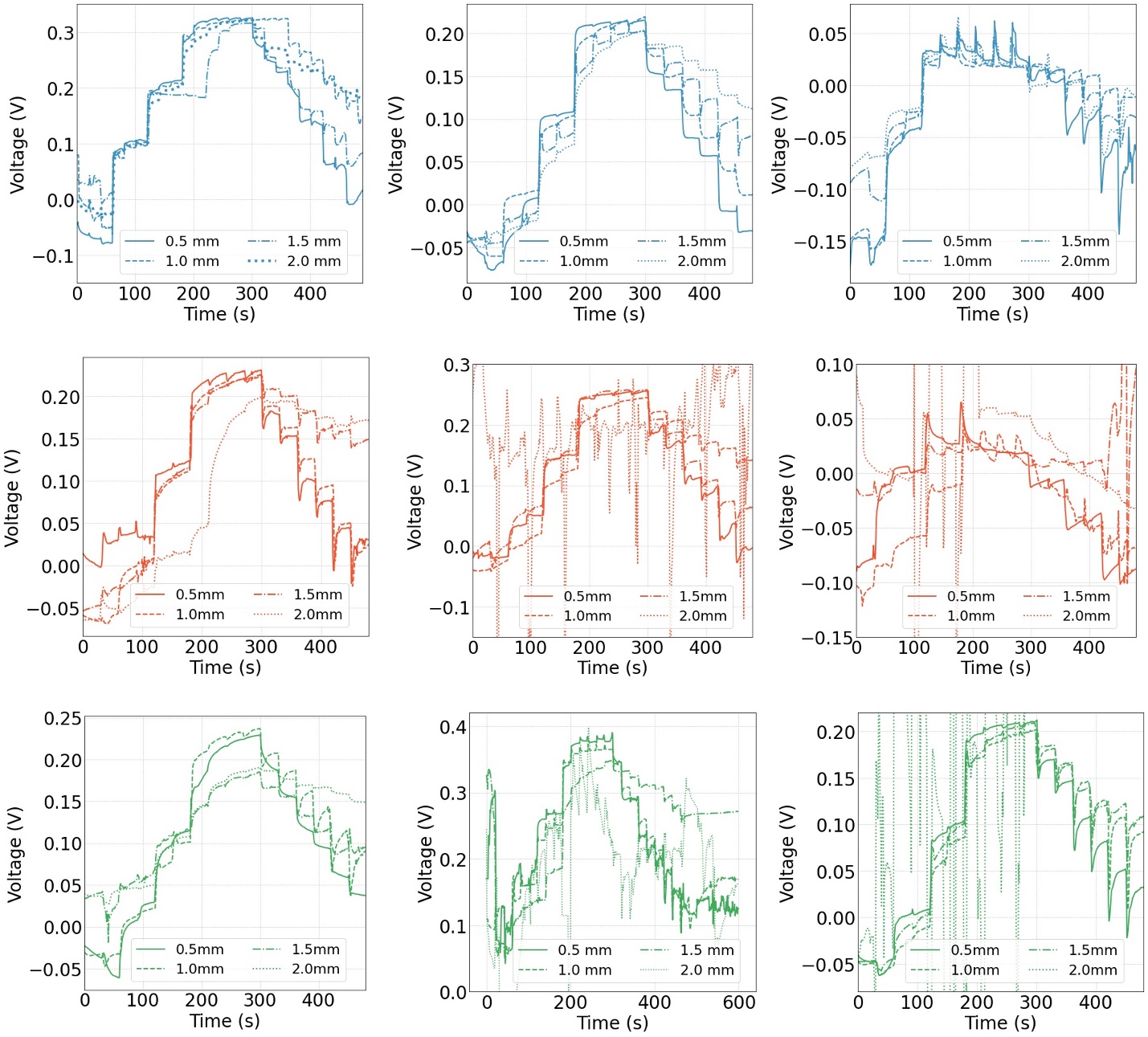
**

**Supplementary Figure 5 | Self-cleaning and distance measurement.** Samples with ascending and descending NaCl concentrations were introduced into the channel to evaluate sensor performance. A total of nine sensors were tested, with three sensors representing each type. Sensors were ranked from best to worst based on performance and arranged accordingly from left to right in the analysis.

Sensor A exhibited the best self-cleaning characteristics and stability across all channel gap sizes, maintaining a stable signal even in the least favorable configurations. In contrast, Sensor B showed good self-cleaning capabilities at smaller gap sizes but demonstrated instability at larger gaps. On one occasion, Sensor B failed to acquire a signal at a 1.5 mm gap. This behavior may be attributed to its wider contact angle observed in the earlier characterization, which creates a stronger hydrophobic/hydrophilic interaction optimized for smaller gaps. Sensor C, while exhibiting the weakest self-cleaning capabilities with the highest slope and upward drift, was able to perform readouts at a 1.5 mm gap in all iterations. However, at a 2 mm gap, Sensor C demonstrated poor contact and inconsistent performance.


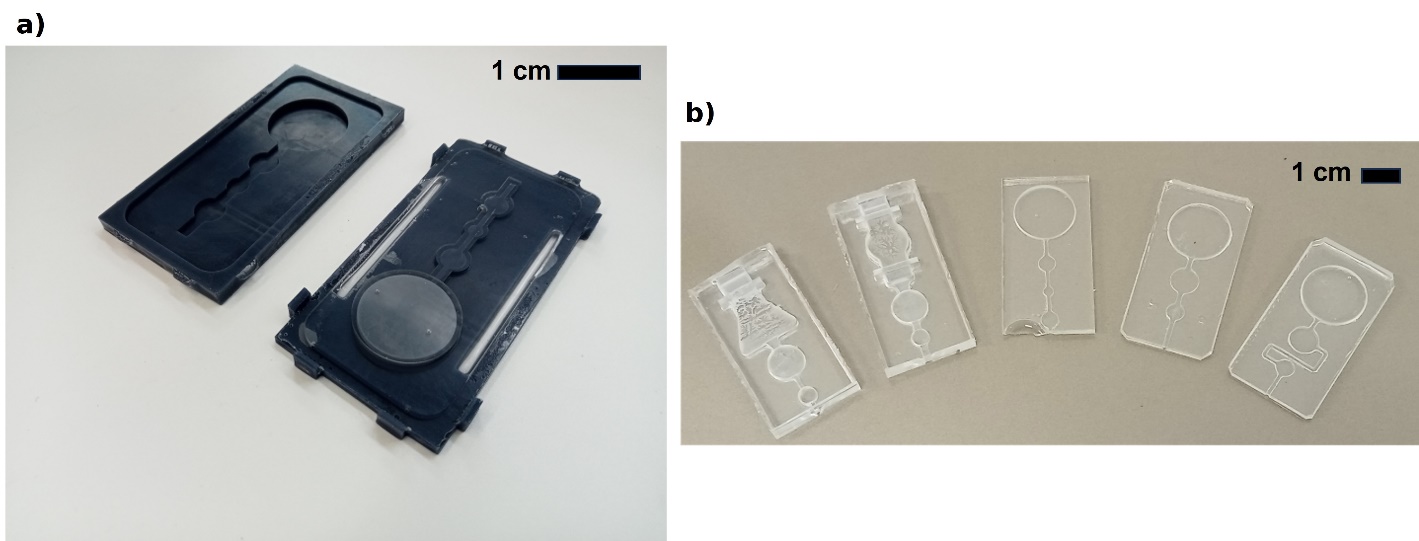


**Supplementary Figure 6 | PDMS device with passive pump. (**a) 3D-Printed Resin Mold for PDMS Casting The 3D-printed resin mold used for casting the PDMS device was cured at 60°C for 24 hours to prevent PDMS curing inhibition. The mold design incorporates microchannels as small as 500 microns in width, enabling precise replication of fine features. Air cavities were integrated into the mold to facilitate the removal of air bubbles from the PDMS using a suction chamber, ensuring uniformity and integrity in the cast device.

(b) Passive Pump Designs for Sweat Flow: Several passive pump designs were evaluated to facilitate active sweat flow within the microchannel. These pumps leverage the cyclical motion of muscles during sporting activities to drive sweat through the channel. However, this approach requires precise placement of the device in strategic locations such as near the elbows or knees, limiting its usability.

To ensure channel closure after removing the PDMS from the mold, a flexible adhesive material is necessary. Currently, Tegaderm is employed due to its compatibility with skin, widespread use in medical applications, and ability to create a flexible seal. While fully cured PDMS is also skin-safe due to its inert properties, establishing a sealing boundary between the sweat inlet port and the skin remains a challenge. This limitation, coupled with dependency on physical constraints and varying skin conditions, led to the passive pump implementation being deemed unsuitable for the study’s objectives.

Although the passive pump concept did not meet the current study's goals, it remains a novel approach worth exploring in future research. Optimizing the sealing mechanism and exploring alternative materials or attachment strategies could enhance the viability of passive pump designs for sweat recirculation and flow control.


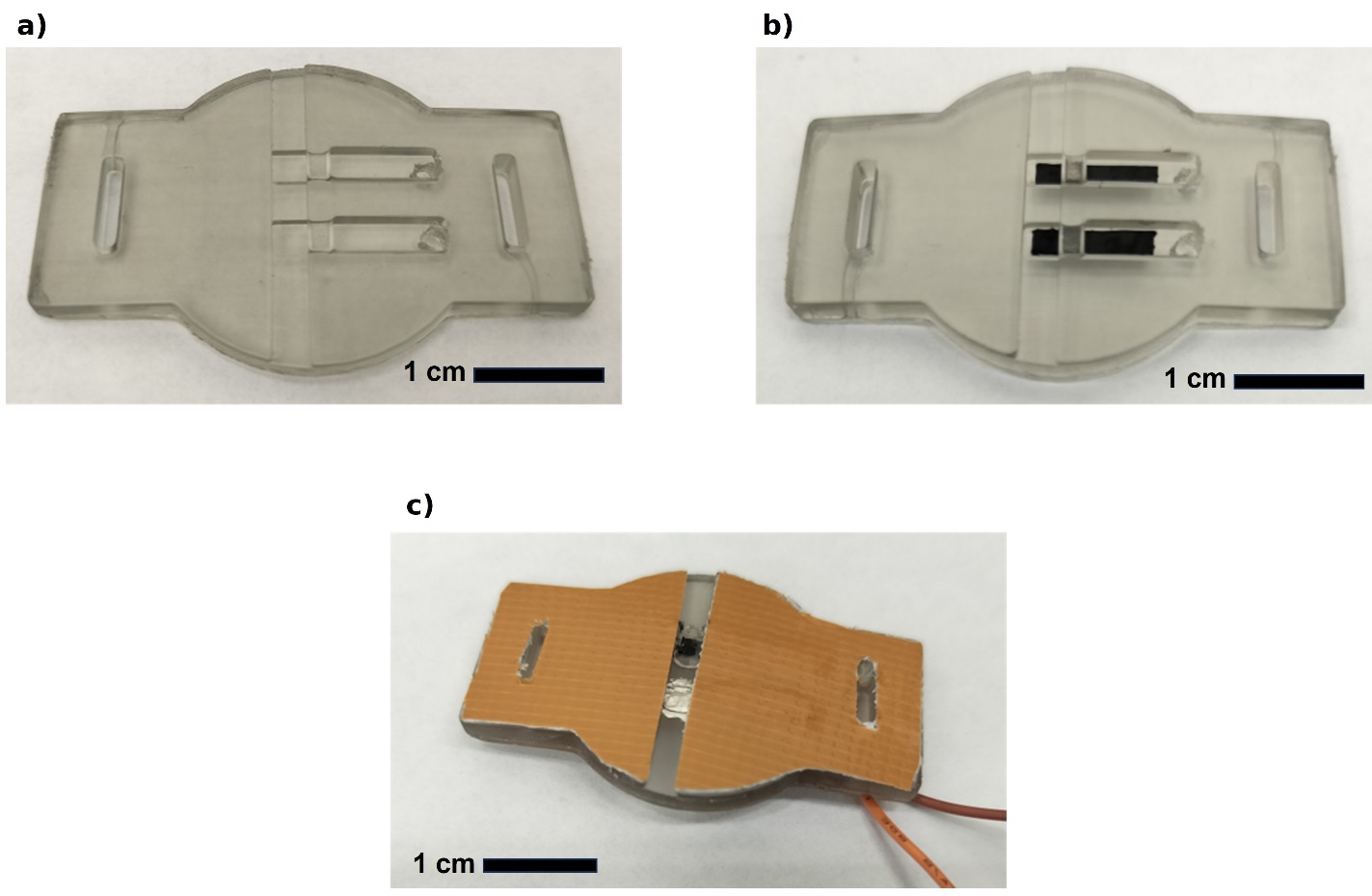


**Supplementary Figure 7 | Flexible UV resin device.** (a) Flexible Resin 3D-Printed Device: A 3D-printed device made from flexible resin was developed to better conform to the body’s natural curvatures. The device design includes:

Slots: Two slots for the placement of sensing and reference electrodes.

Channel: A channel with a fixed air gap for sweat flow.

Attachment Mechanism: Two holes designed to secure the device with an elastic band, allowing for adjustable fit and stability during motion.

(b) Electrode Insertion and Sealing: The CNT electrodes were inserted into the designated slots and subsequently sealed with an elastomer sealant. This seal prevents sweat from leaking outside the sensing channel, maintaining a controlled environment for accurate measurement.

(c) Skin-Interfacing Tape and Electrode Assembly: To avoid direct contact between the device and the skin, interfacing tape was applied. This tape also helped enhance the sealing between the skin and the device. The reference electrode (Ag/AgCl) is visible as a silver material, while the transparent ISM allows the CNTF substrate color to show through. Connections between the electrodes and cables were established using Ag/AgCl ink, enabling a "cold soldering" process that ensures electrical connectivity without traditional soldering techniques.

The flexible nature of the device presented challenges, such as minor leaks along the channel, which compromised signal integrity. Additionally, the straight-line channel design resulted in overly fast sweat flow, leading to bubble formation and signal inaccuracies. Despite these issues, the device’s simplicity, ease of electrode mounting, and adaptability to the body provided valuable insights and served as a foundational concept for the development of the final design.


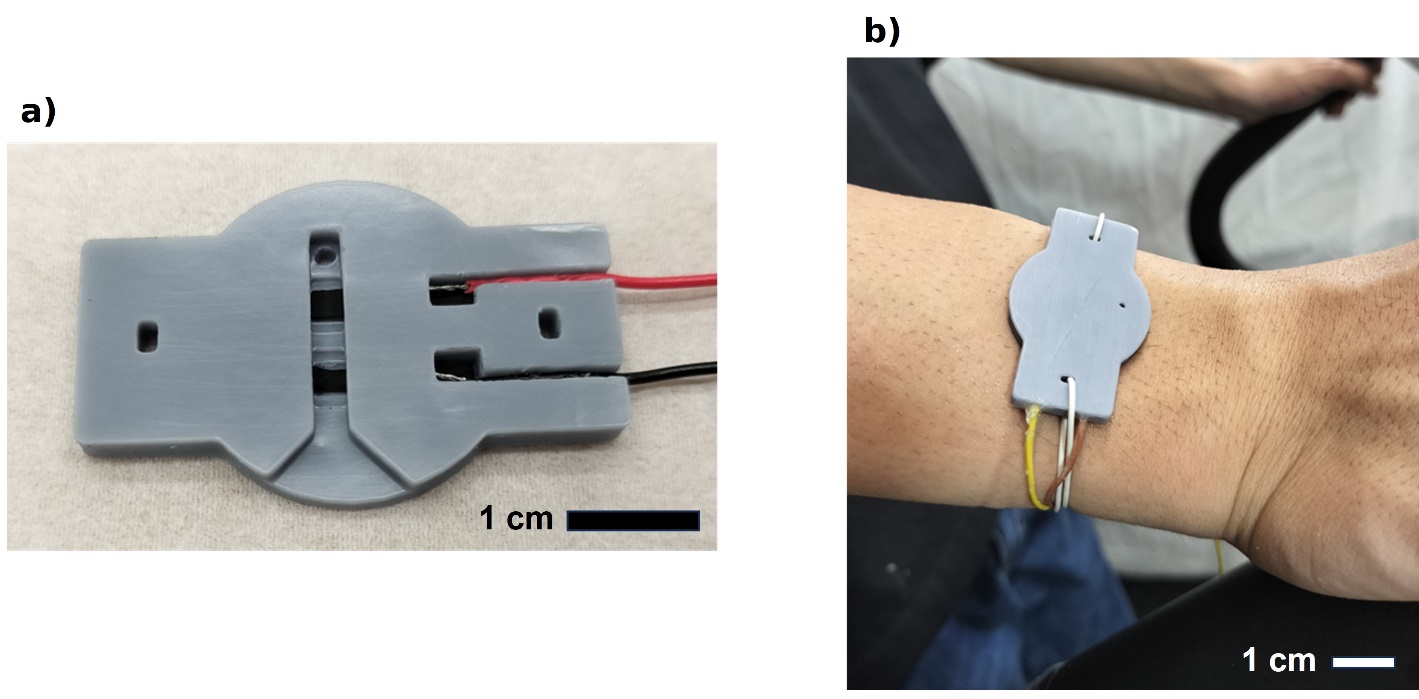


**Supplementary Figure 8 | Device using hard resin.** (a) Enhanced 3D-Printed Electrochemical Sensor Design: The improved 3D-printed device integrates an electrochemical sensor with sealed electrodes for optimal performance. Key features include:

Three-Dimensional Channel Path: The channel now follows a 3D path, with the sweat inlet directed toward the skin and the outlet positioned perpendicularly. This configuration generates a flow-limiting force while facilitating sweat transport.

Funnel-Shaped Inlet: The inlet has been redesigned with a funnel shape to capture surrounding sweat more efficiently and guide it into the channel.

Material Enhancement: Constructed from hard UV resin, the channel’s sealing issues were fully resolved, ensuring long-term stability of the measurements. This improvement minimizes leakage and allows stable signal acquisition. Occasionally occurring small air bubbles can now be easily filtered out during post-processing, preserving data accuracy.

(b) Usability and Wearability: The device is securely positioned on the dorsal wrist using an elastic rubber band, optimizing comfort for extended use. This positioning resembles commercial wearables like fitness bands or smartwatches, allowing users to easily remove the device while maintaining full functionality.

Connection: The device is linked to a desktop potentiostat via two cables for real-time electrochemical readouts.

Comfort Optimization: The tension of the elastic band has been fine-tuned to strike a balance between secure placement and user comfort, ensuring usability during prolonged activity.

This refined design enhances practicality and performance, overcoming challenges in flow management and leakage while offering a user-friendly interface suitable for real-world applications.


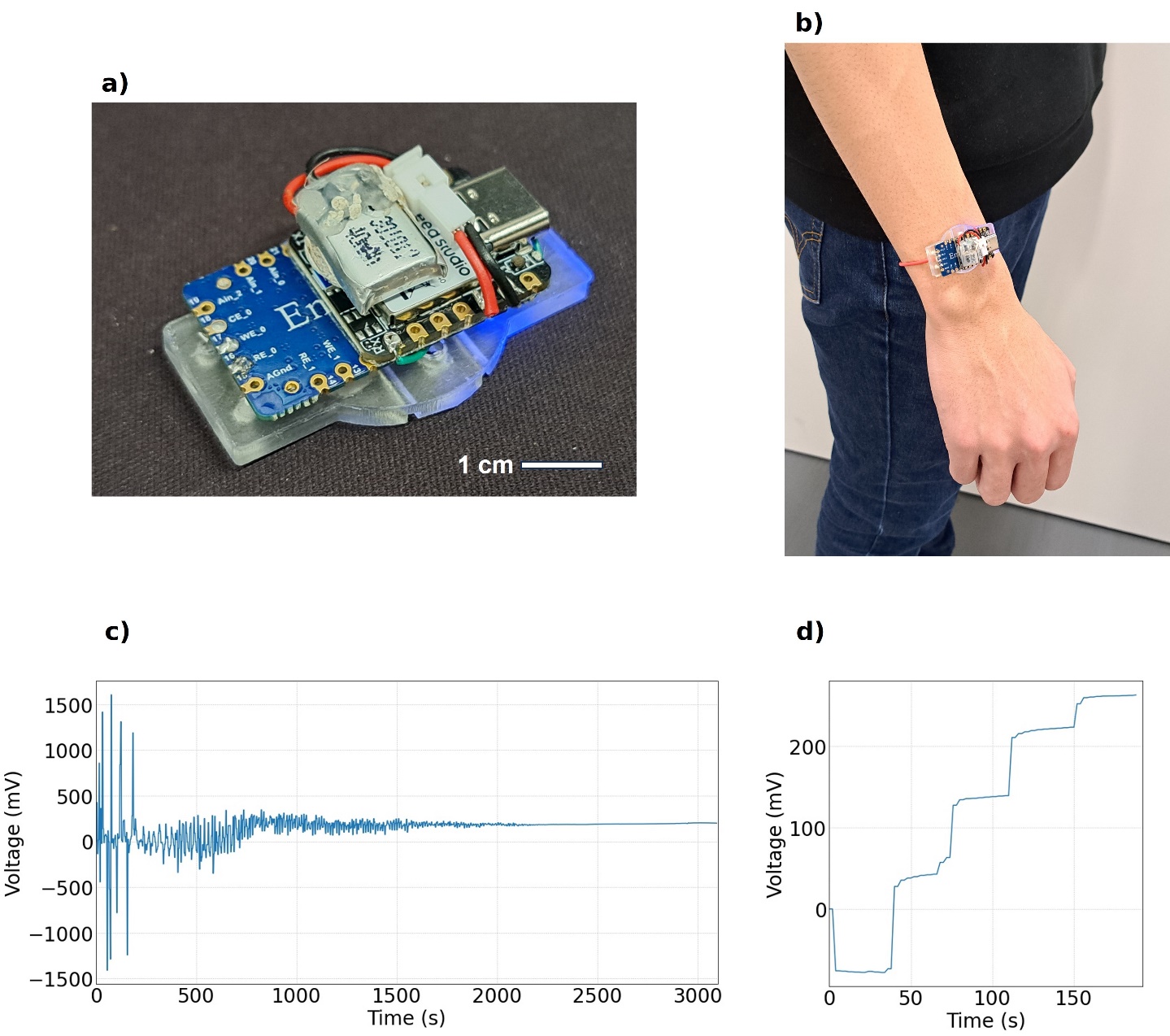


**Supplementary Figure 9 | Miniaturized wireless potentiostat.** (a) Miniaturized potentiostat: The system incorporates an EmStat Pico Module for open circuit potential (OCP) measurements, coupled with an nRF5240 Bluetooth microcontroller for wireless communication with a smartphone for real-time data monitoring and processing. It operates on a 3.7 V, 40 mAh LiPo battery, enabling continuous usage for up to 1 hour, with a sampling rate of 30 samples per minute.

(b) Device positioning: The wearable device is designed to be worn like a smartwatch and can accommodate additional sensors, such as pulse oximeters and heart rate monitors, utilizing the same sensing area for multifunctional applications.

(c) Real-time running experiment: During on-body testing, the device maintained stable performance with minimal noise. Signal stabilization occurred once the sweat channel was filled, confirming the system’s robustness during dynamic conditions.

(d) Response time and sensitivity: The system’s performance is comparable to laboratory potentiostat. Sequential application of NaCl solutions (0 M to 10−1 M) dissolved in deionized water demonstrated reliable response times and sensitivity, validating its capability for wireless operation.
